# Engineered Cyanobacteria‐Hydrogel Microecological System for Oxygen Reprogramming Synergizes Immunomodulation With Anabolism in Osteoarthritis Therapy

**DOI:** 10.1002/advs.77248

**Published:** 2026-08-31

**Authors:** Lerong Yang, Chong Shen, Hongjun Huang, Siyi Liu, Zeyu Huang, Licheng Xi, Huan Cao, Li Zheng, Zainen Qin

**Affiliations:** ^1^ Guangxi Key Laboratory of Regenerative Medicine Guangxi Engineering Center in Biomedical Materials For Tissue and Organ Regeneration International Joint Laboratory On Regeneration of Bone and Soft Tissues Collaborative Innovation Center of Regenerative Medicine and Medical Bioresource Development and Application Co‐Constructed By the Province and Ministry The First Affiliated Hospital of Guangxi Medical University Guangxi Medical University Nanning China; ^2^ Department of Orthopaedics Affiliated Hospital of Guilin Medical University Guilin Guangxi China; ^3^ Life Sciences Institute Guangxi Medical University Nanning China; ^4^ National Engineering Research Center for Biomaterials College of Biomedical Materials Sichuan University Chengdu China; ^5^ Yida collaborative Medical Technology Co., Ltd. Guangxi Medical University Nanning China

**Keywords:** cartilage anabolism, cyanobacteria, osteoarthritis, oxygen metabolism reprogramming, photocontrollale hydrogel, synovial inflammation

## Abstract

Osteoarthritis remains a major clinical challenge due to its complex pathophysiology involving chronic inflammation and progressive cartilage degradation. Current treatments largely fail to address the dual demands of synovial anti‐inflammation and cartilage anabolism, particularly under the critical yet often overlooked influence of oxygen zonation within articular tissues. In this study, we propose a novel microorganism oxygen metabolism engineering strategy using an injectable cyanobacteria‐laden hydrogel (R‐alga) that enables light‐dependent dynamic oxygen regulation to mimic native physiological oxygen gradients. Under infrared ray (red) light irradiation, cyanobacteria within R‐alga efficiently produce oxygen via photosynthesis, specifically targeting and alleviating hypoxia in the inflamed synovial region. This process significantly suppresses the aberrant expression of HIF‐1α in macrophages, ameliorates their metabolic dysfunction, and effectively scavenges ROS, ultimately inducing macrophage polarization toward an pro‐regenerative phenotype. In the absence of light, cyanobacteria within R‐alga actively consume oxygen through respiration, creating and maintaining a physiologically relevant hypoxic microenvironment in the cartilage defect area. This environment stabilizes HIF‐1α expression in chondrocytes, activates the HIF‐1α‐DOT1L signaling axis, and significantly enhances the biosynthesis of key cartilage matrix components, thereby facilitating cartilage repair. Collectively, the microorganism oxygen metabolismengineering strategy offers a breakthrough for precise control of the osteoarthritis microenvironment and therapy.

## Graphical abstract

An injectable cyanobacteria‐laden hydrogel enables light‐dependent oxygen metabolism engineering for osteoarthritis therapy. Photosynthetic oxygen release under red light irradiation suppresses synovial inflammation via HIF‐1α inhibition and M2 macrophage polarization, while respiratory oxygen consumption in darkness stabilizes chondrocyte HIF‐1α to activate epigenetic‐metabolic reprogramming and enhance cartilage matrix biosynthesis, achieving synergistic therapeutic efficacy.

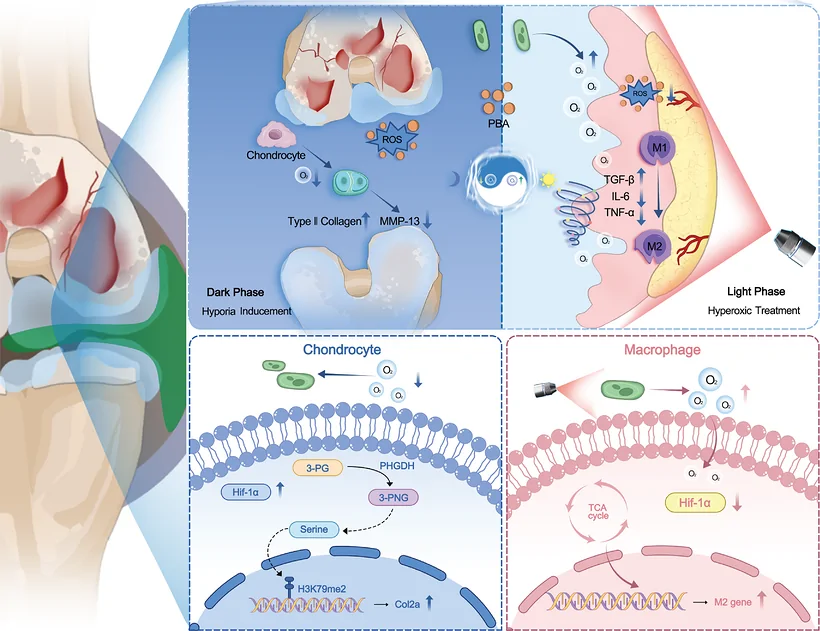

## 1 Introduction

Osteoarthritis (OA) is a chronic degenerative disease primarily characterized by progressive articular cartilage destruction, often accompanied by synovial inflammation, osteophyte formation, and joint dysfunction, and has becoming one of the leading causes of disability worldwide [1, 2, 3]. The escalating prevalence of OA due to an aging global population underscores the urgent need for innovative and effective therapeutic strategies to address its substantial social burden.

Generally, the pathological progression of OA is intricately linked to a vicious cycle of microenvironment imbalance within the articular cavity [4, 5, 6]. Synovial inflammation, in particular, is recognized as a critical driver of cartilage degradation [7, 8]. Aberrant polarization of synovial macrophages toward the pro‐inflammatory M1 phenotype leads to the excessive release of inflammatory cytokines, chemokines, and reactive oxygen species (ROS), initiating an inflammation‐degradation cascade that further damages the cartilage matrix [9, 10, 11, 12]. In brief, current clinical strategies for OA mainly rely on non‐steroidal anti‐inflammatory drugs (NSAIDs) as well as intra‐articular injections of corticosteroids or hyaluronic acid. While these approaches provide short‐term symptomatic improvement, they fail to reverse structural cartilage injury or break the core inflammation‐oxidation cycle underlying OA progression [13, 14]. In addition, long‐term administration of these agents is accompanied by considerable adverse events. For individuals with end‐stage OA, joint replacement surgery remains the only definitive option; nevertheless, it is an invasive and costly intervention associated with inherent perioperative and postsurgical complications [15]. Therefore, the development of innovative therapeutic agents that target and interrupt the inflammation‐oxidation cycle at its source is urgently needed for more effective OA management.

However, the microenvironment within the articular cavity is complex and highly heterogeneous, functioning as a confined niche where distinct regions exhibit opposing oxygen requirements. Anatomically, the joint's limited vascularity dictates that oxygen partial pressure within the cartilage typically ranges from 1% to 5%, forming a decreasing gradient from the superficial to the deep zones [16]. Within this physiologically hypoxic environment, hypoxia‐inducible factor‐1α (HIF‐1α) acts as a critical mediator of cellular oxygen sensing and adaptation [17, 18, 19, 20, 21, 22]. Yet, during the progression of OA, HIF‐1α exerts a paradoxical, tissue‐specific effect. In the synovium, the aberrant proliferation of fibroblasts and substantial infiltration of immune cells, particularly macrophages, significantly elevate local oxygen consumption. Concurrently, dysfunctional angiogenesis fails to meet this metabolic demand, leading to the pathological accumulation of HIF‐1α. This accumulation drives macrophage polarization toward the pro‐inflammatory M1 phenotype, suppresses the tricarboxylic acid (TCA) cycle in favor of glycolysis, and promotes the release of ROS, thereby exacerbating inflammation [23, 24, 25, 26]. Clinically, synovial HIF‐1α levels correlate positively with OA severity, and targeted deletion of HIF‐1α in macrophages notably mitigates synovial inflammation and cartilage degradation [27]. Conversely, under pathological hypoxic conditions, HIF‐1α expression is downregulated in chondrocytes. This suppression impairs cellular stress adaptation, induces a metabolic shift toward anaerobic glycolysis, exacerbates ROS accumulation, and upregulates catabolic enzymes such as matrix metalloproteinases (MMPs), ultimately driving cartilage degeneration [21, 28, 29]. Therefore, restoring a physiological hypoxic state and stabilizing HIF‐1α expression have emerged as crucial therapeutic strategies for cartilage repair.

Currently, various strategies, such as exogenous oxygen‐supplying materials (nano‐oxygen carriers, enzyme‐catalyzed oxygen release) or oxygen‐consuming systems (hypoxia‐mimicking or oxygen‐scavenging materials) [18, 28, 30, 31, 32], have been developed, but predominantly focus on a single mode of action and lack the capability for precision control of the intra‐articular microenvironment. Cyanobacteria, as an ancient photosynthetic microorganisms, possess unique bidirectional oxygen metabolism characteristics. They produce oxygen through photosynthesis under light and consume oxygen through respiration in darkness. This photo‐responsive oxygen metabolism endows them with the potential to act as smart oxygen factories [33, 34, 35, 36, 37]. Previous studies have shown that cyanobacteria can modulate local oxygen levels, for instance, by promoting macrophage M2 polarization or mimicking hypoxia to protect the myocardium [30, 38]. However, their potential for dual‐targeted synergistic therapy for OA through differentiated oxygen metabolism reprogramming has not been reported.

**FIGURE 1:**
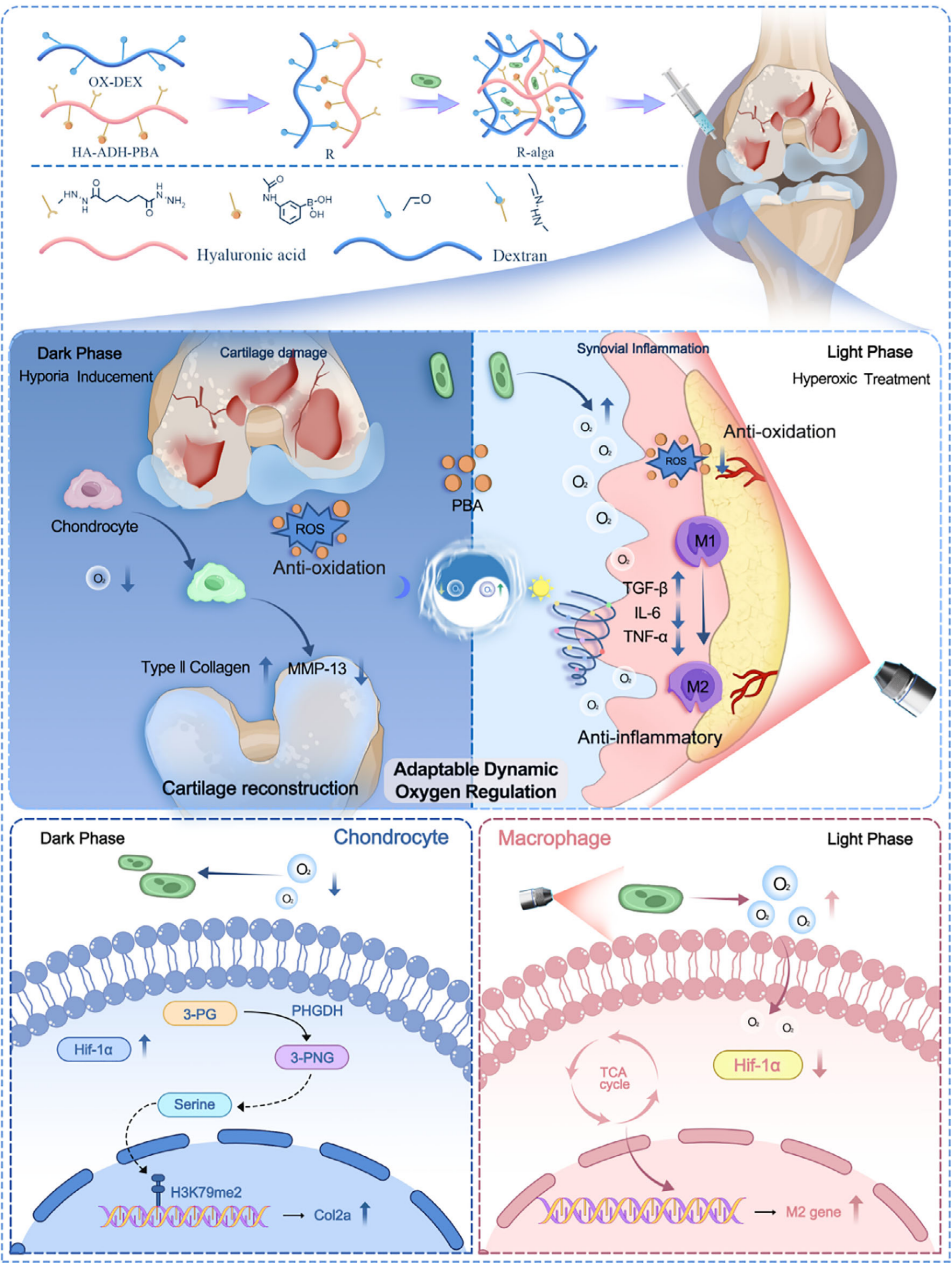
Schematic diagram of the design, preparation and OA therapeutic mechanism of photocontrollable R‐alga hydrogel, where R‐alga hydrogel enables dual‐targeted synergistic therapy through differentiated oxygen metabolism reprogramming.

Hence, this study proposes a microorganism oxygen metabolism engineering therapeutic strategy through constructing a smart photocontrollable cyanobacteria‐based hydrogel (R‐alga) platform (Figure 1). In particular, 3‐aminophenylboronic acid (PBA) and adipic dihydrazide (ADH)‐grafted hyaluronic acid (HA‐ADH‐PBA) was reacted with oxidized dextran (OX‐DEX) through Schiff base formation, generating an injectable hydrogel, in which cyanobacteria as oxygen‐regulating engines are integrated and confer adaptive dynamic oxygen regulation capabilities to hydrogel. Under Red irradiation, cyanobacteria initiate photosynthesis, directionally supplying oxygen to the synovial inflammatory region, effectively alleviating local hypoxia, inhibiting abnormal HIF‐1α activation, and promoting macrophage polarization towards the pro‐regenerative M2 phenotype, thereby suppressing inflammatory responses. Conversely, in the absence of Red light, cyanobacteria actively consume oxygen through respiration, maintaining a physiologically hypoxic microenvironment in the cartilage damage area. This stabilizes chondrocyte HIF‐1α activity, activates HIF‐1α‐DOT1L and other downstream protective signaling pathways, and significantly enhances matrix synthesis and cartilage repair capabilities. This system not only enables synergistic regulation of anti‐inflammatory and pro‐repair pathological processes within a single therapeutic platform, but also overcomes the limitations of traditional materials in responding to complex microenvironmental changes. Moreover, the introduction of PBA groups further enhances hydrogel ROS scavenging ability [39, 40, 41, 42], establishing an antioxidant microenvironment conducive to tissue repair. The platform also exhibits excellent injectability, tissue adhesiveness, self‐healing properties, and biocompatibility, demonstrating promising prospects for intra‐articular application. Collectively, this study not only constructs an oxygen‐responsive smart material system based on live microorganisms, but also pioneeringly proposes microorganism oxygen metabolism engineering as a novel therapeutic paradigm, addressing the long‐standing challenge of microenvironmental heterogeneity in OA treatment and providing a solid foundation for achieving systemic synergistic therapy involving efficient anti‐inflammation, precise immune modulation, and cartilage regeneration, thereby demonstrating broad clinical translation potential.

## 2 Results and Discussion

### 2.1 Preparation and Characterization of R‐alga Hydrogel

**FIGURE 2:**
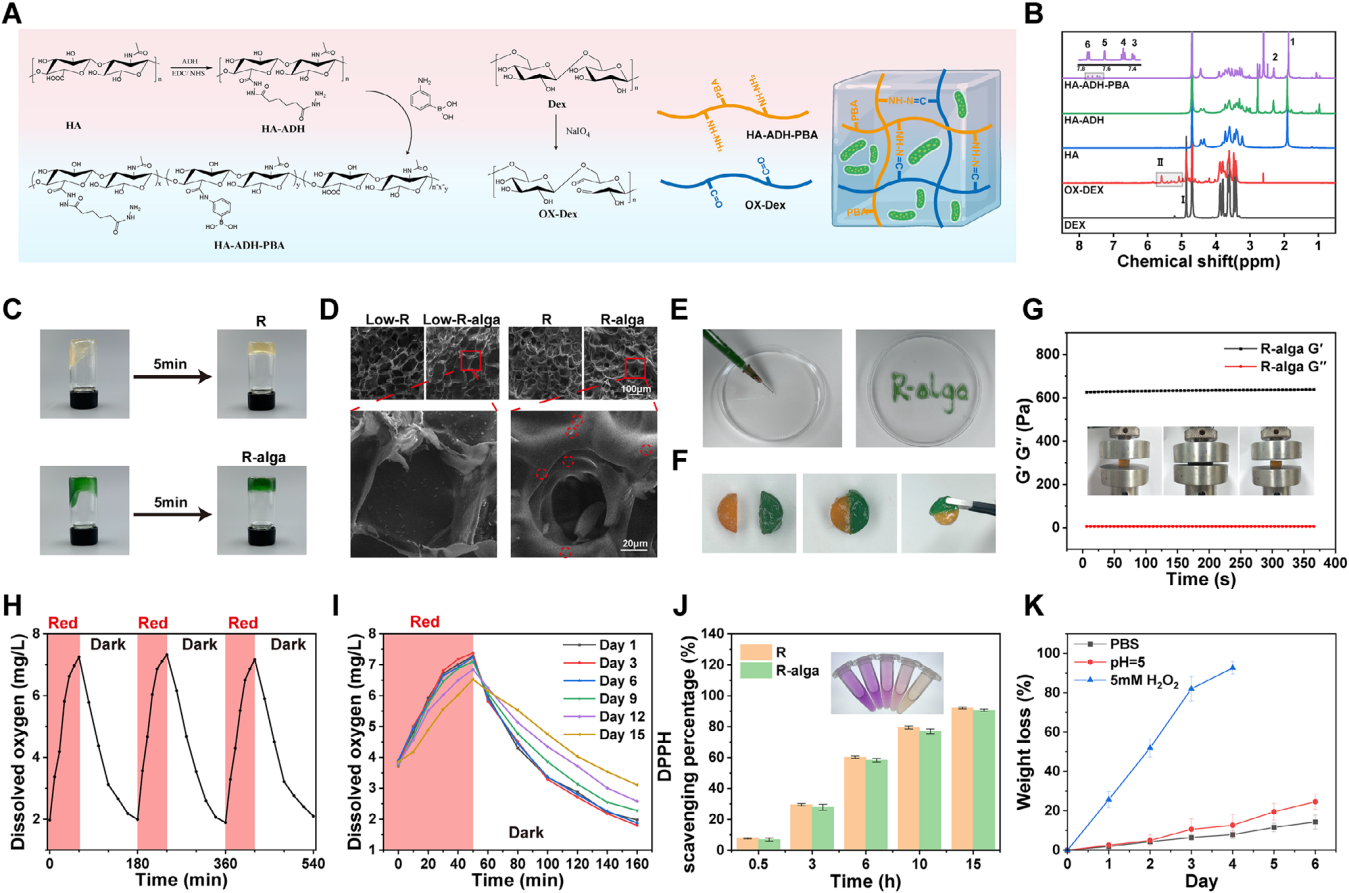
Construction and characterization of R‐alga hydrogel. (A) Structure diagram of R‐alga hydrogel synthesis; (B) NMR hydrogen spectra of DEX, OX‐Dex, HA, HA‐ADH, HA‐ADH‐PBA; (C) Inversion test of R hydrogel and R‐alga hydrogel; (D) SEM pictures of different hydrogels, where red circles indicate cyanobacteria (1×10^7^ cells/mL); (E) Injectability of R‐alga hydrogel; (F) Photos of hydrogel before and after self‐healing; (G) storage modulus and loss modulus of R‐alga hydrogel over time; (H) Cyclical oxygen release and consumption of R‐alga hydrogel; (I) Oxygen release and consumption profiles of R‐alga hydrogel over 15 days; (J) Free radical scavenging ability of hydrogel; (K) Degradation curves of R‐alga hydrogel under different conditions. Data are expressed as mean ± SD (*n* = 3).

To address the key pathological features of hypoxia and oxidative stress within the OA joint cavity, as well as the challenge posed by microenvironmental oxygen heterogeneity, this study developed a photo‐responsive, oxygen‐dynamically regulating injectable R‐alga hydrogel. First of all, the R‐alga hydrogel precursors were synthesized and natural polymers HA and dextran were selected as the base materials. Specifically, the ^1^H NMR spectrum of the obtained HA‐ADH‐PBA exhibited new peaks at 7.74–7.37 ppm (aromatic protons) and at 2.38–2.27 ppm (methylene protons of ADH), suggesting the incorporation of both functional moieties. Meanwhile, dextran was oxidized with sodium periodate to obtain aldehyde‐functionalized dextran (OX‐DEX), and the ^1^H NMR spectrum of OX‐DEX displayed new distinctive signals at 5.0–6.0 ppm (Figure 2A,B). Then, FTIR spectroscopy suggested the dynamic Schiff base bond formation between HA‐ADH‐PBA and OX‐DEX with characteristic peak at 1652 cm^−^^1^ (C = N stretch) (Figure S1), endowing the formed R hydrogel with good injectability and self‐healing properties [43, 44].

Following which, alga was incorporated into R hydrogel to obtain R‐alga hydrogel, and its physicochemical properties and tunable oxygen dynamics were comprehensively characterized. The R‐alga hydrogel formed within 5 min, displaying a uniform, interconnected porous structure that enhanced water retention and supported cyanobacterial growth (Figure 2C,D). It demonstrated excellent injectability via fine needles (ID = 0.33 mm) (Figure 2E) and rapid self‐healing within 5 min (Figure 2F), making it highly suitable for minimally invasive applications such as intra‐articular delivery [45, 46, 47]. Rheological tests confirm the R‐alga hydrogel exhibited a stable network (G′> G″), withstanded large strains (>100%), and showed shear‐thinning capacity. Compressive strength reached 0.314 ± 0.028 MPa, indicating high mechanical robustness (Figure 2G and Figures S2–S4).

The most important feature of the R‐alga hydrogel lies in the tunable oxygen dynamics conferred by the encapsulated live cyanobacteria (1×10^7^ cells/mL of cyanobacteria in R‐alga hydrogel). The analysis using a dissolved oxygen electrode confirmed that under red light (630 nm) irradiation, the hydrogel continuously released oxygen through cyanobacterial photosynthesis, while exhibiting significant oxygen consumption via respiration in dark conditions (Figure S5). Furthermore, Cyanobacteria in the R Gel cultured after various storage durations all grew well, with no significant differences in cell numbers (Figures S6, S7). More importantly, the R‐alga hydrogel stably maintained its oxygen release and consumption capabilities over multiple light/dark cycles (Figure 2H). Even after 15 days of storage, their photosynthetic and respiratory capacities showed no significant decline (Figure 2I). All these results demonstrated that cyanobacterial encapsulated in R possessed satisfying stability of photosynthetic O_2_ generation. Furthermore, DPPH radical scavenging experiments showed that the R‐alga hydrogel possessed significant antioxidant capacity, with the scavenging rate increasing linearly with time, reaching as high as around 90% after 15 h (Figure 2J), confirming its potential ROS scavenging ability in the pathological OA environment. The ROS responsibility of hydrogel was also confirmed in degradation test (Figure 2K), which is consistent to previous report [71]. Collectively, based on the photo‐controlled oxygen dynamic regulation of cyanobacteria and ROS scavenging capabilities, the R‐alga hydrogel, integrating excellent injectability, elasticity, and self‐healing properties, provides a novel and highly promising therapeutic strategy for intra‐articular microenvironment modulation and repair in OA.

### 2.2 Biocompatibility and Antioxidant Activity of R‐alga Hydrogel

Building upon the above outstanding physicochemcial of R‐alga hydrogel, we further evaluated its cytocompatibility and intracellular antioxidant capacity. First, the cytotoxicity of the hydrogel was evaluated using CCK‐8 and cellular DNA content assays on primary rat chondrocytes and mouse macrophage cell lines (RAW 264.7). (Figures S8, S9). This finding was corroborated by calcein‐AM/PI double staining assays, which showed negligible PI‐positive (dead) cells even under Red light irradiation after co‐culture with R‐alga hydrogel (Figure S10). Given that excessive ROS generation and subsequent oxidative stress are the core pathological mechanism in chondrocyte damage and degeneration in OA [48, 49], we specifically examined the intracellular antioxidative efficacy of R‐alga hydrogel. To mimic the pathological oxidative microenvironment, rat chondrocytes were exposed to 500 µM H_2_O_2_. Both fluorescence microscopy imaging (Figure S11a) and flow cytometry quantitative analysis (Figure S11b) clearly revealed that the R‐alga hydrogel significantly scavenged intracellular ROS and protect chondrocytes from H_2_O_2_‐induced oxidative damage. Overall, these results demonstrate the good biocompatibility and potential to mitigate oxidative stress‐mediated cell injure of as‐prepared R‐alga hydrogel under OA condition.

### 2.3 R‐alga Hydrogel Promotes Metabolic and Phenotypic Reprogramming of Osteoarthritic Macrophages

**FIGURE 3:**
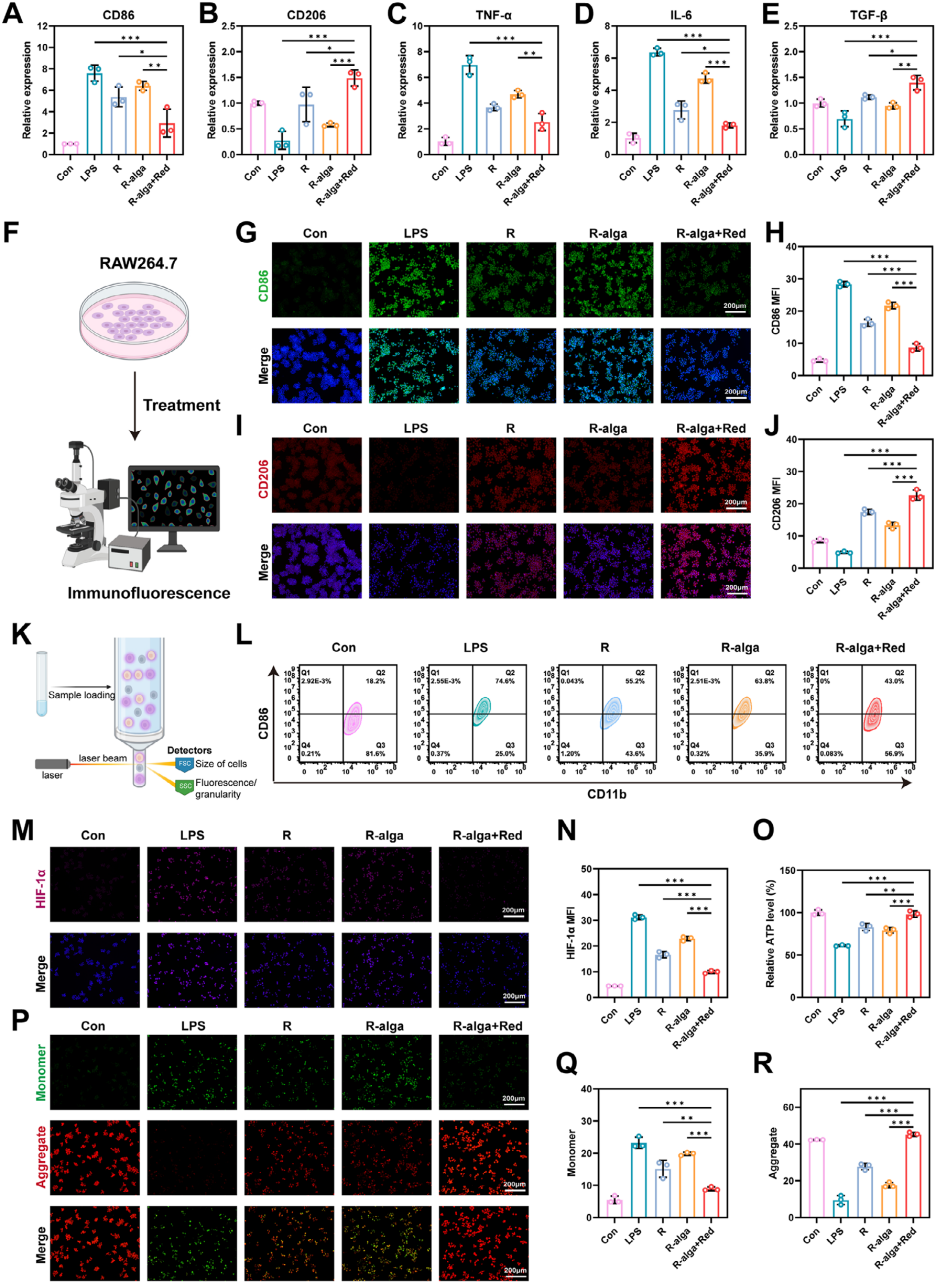
Phenotypic and metabolic reprogramming of osteoarthritic macrophages via photo‐stimulated R‐alga hydrogel. (A–E) Gene expression of M1 macrophage markers (CD86, TNF‐α, IL‐6) and M2 macrophage markers (CD206, TGF‐β) after different treatments; (F) schematic image of RAW264.7 under different treatments; (G–J) Immunofluorescence staining images and corresponding statistical results of CD86^+^ (G‐H) and CD206^+^ (I, J) macrophages after different treatments; (K) schematic image of flow cytometry analysis of RAW264.7 cells under different treatments; (L) Flow cytometry results of CD86^+^ macrophage after different treatments; (M,N) Immunofluorescence staining and corresponding statistical results of HIF‐1α in macrophages treated with different groups; (O) Relative fluorescence intensity statistics of ATP in macrophages after different treatments; (P–R) Fluorescence staining and corresponding statistical results of JC‐1 in macrophages after different treatments. Data are expressed as mean ± SD (*n* = 3). *p < 0.05, **p < 0.01, ***p < 0.001.

As well‐established, hypoxia conditions and the abundance of pro‐inflammatory M1 macrophages in the OA microenvironment critically contribute to disease progression [50]. Hence, we further systematically evaluated the capacity of R‐alga hydrogel to induce macrophage phenotypic reprogramming‐ particularly under Red light irradiation—and to elucidate the underlying mechanism. In detail, RAW264.7 macrophages were cultured in a hypoxia incubator, and lipopolysaccharide (LPS) was employed to polarize RAW264.7 toward M1‐like phenotype. The resulting M1‐like macrophages were subsequently treated with R‐gel, R‐alga gel, and R‐alga + Red, respectively, to evaluate their immunomodulatory effects. First of all, oxygen probe [Ru(dpp)_3_] Cl_2_ was used to evaluate the intracellular oxygen levels. As shown in Figure S12, red fluoresence signals could be detected in cells due to the hypoxic environment, while decreased signals was observed in R‐alga + Red group due to the oxygen production by alga. Moreover, the qRT‐PCR analysis showed that LPS stimulation significantly upregulated the expression of M1‐like markers (CD86, TNF‐α, IL‐6) while downregulated M2‐like markers (CD206, TGF‐β) at the transcriptional level (Figure 3A–E). Notably, R‐alga + Red treatment significantly reversed this polarization imbalance. In contrast, R‐alga treatment alone (without Red light irradiation) showed modest effects on downregulating CD86 and CD206 upregulation, presumably due to oxygen consumption by cyanobacterial respiration in the absence of light. Furthermore, immunofluorescence staining and flow cytometry detection showed a pronounced increase in CD86^+^ signals and reduction in CD206^+^ signals following LPS stimulation. Conversely, R‐alga + Red treatment significantly reduced CD86^+^ fluorescence intensity significantly and enhanced CD206^+^ expression (Figure 3G–L and Figure S13), indicating macrophage reprogramming from M1‐like to M2‐like phenotype. Although R hydrogel treatment, itself alone also induced certain reprogramming effect, its efficacy was weaker than that of R‐alga + Red treatment, which may be related to its lack of photo‐tunable oxygen supply despite possessing ROS‐scavenging capacity. Moreover, compared to the R group, HIF‐1α expression significantly elevated in the R‐alga group but significantly suppressed in the R‐alga + Red group (Figure 3M,N), indicating that the enhanced macrophage reprogramming effect of R‐alga + Red involves synergistic inhibition of the HIF‐1α pathway and ROS scavenging.

Further investigation delved into the cellular metabolism, which is fundamental to macrophage function and phenotype maintenance. Using an ATP assay kit, we observed that R‐alga + Red treatment significantly increased intracellular ATP levels in LPS‐stimulated macrophages (Figure 3O), indicating effective alleviation of hypoxia and re‐establishment of metabolic homeostasis in macrophages. Mitochondrial membrane potential (MMP), a key indicator reflecting mitochondrial function and aerobic metabolic status, was assessed using JC‐1 probe [51]. LPS stimulation led to a decrease in MMP with enhanced green monomer fluorescence. Both R and R‐alga + Red treatments promoted MMP recovery, as evidenced by enhanced red aggregate fluorescence (Figure 3P–R). These results confirmed the ability of R‐alga + Red treatment to rescue mitochondrial dysfunction and restore aerobic metabolism in M1‐polarized macrophages. In summary, the photo‐controllable R‐alga hydrogel can effectively promote macrophages re‐polarization from pro‐inflammatory M1‐like to pro‐regenerative M2‐like phenotype through a synergistically mechanism involving attenuation of local hypoxia, ROS effective scavenging, and recovery of mitochondrial function and energy metabolism, providing a favorable immune microenvironment conducive to tissue repair in OA.

### 2.4 R‐alga Hydrogel Potentiates Anabolism in Osteoarthritic Chondrocytes

**FIGURE 4:**
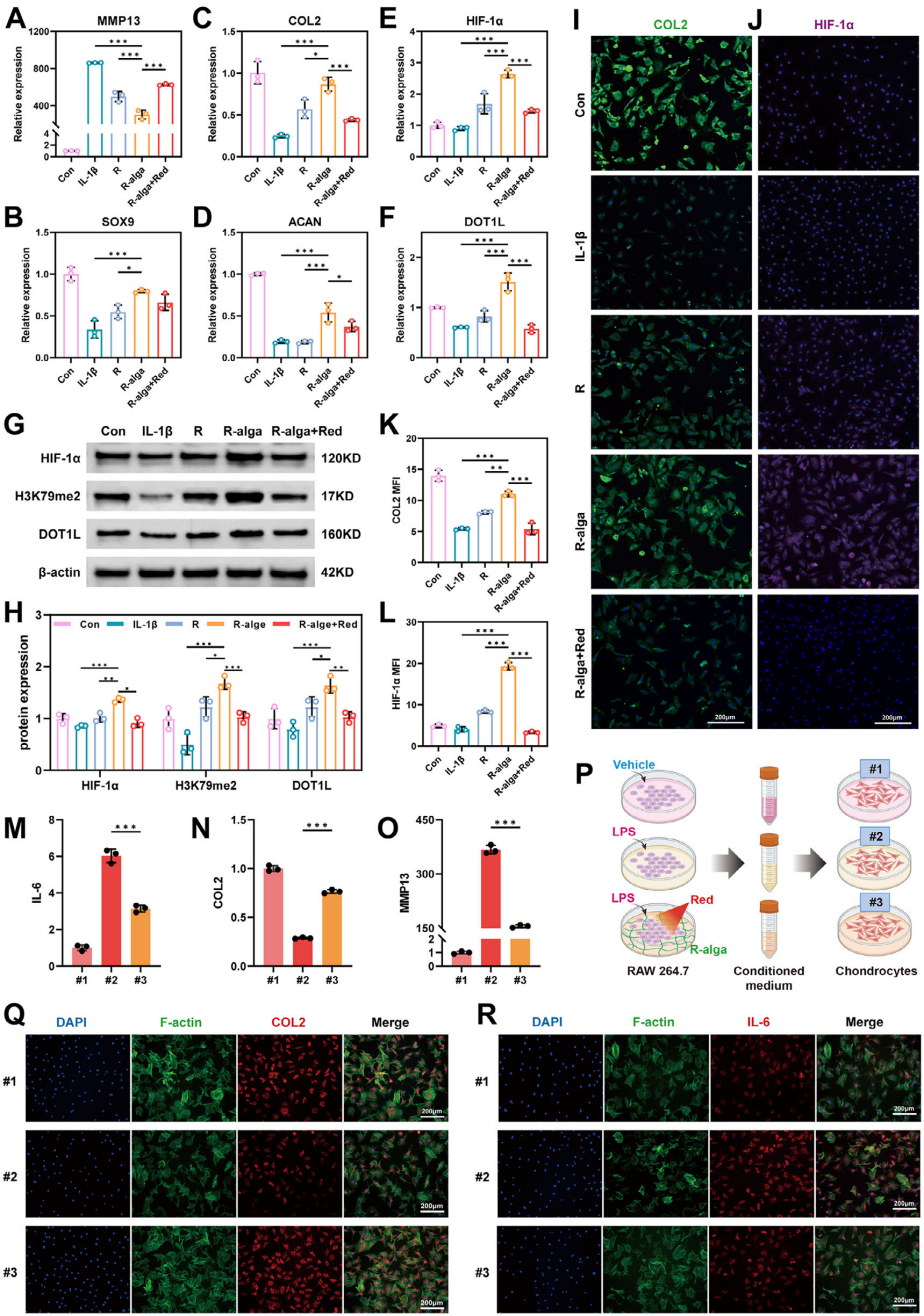
R‐alga Hydrogel Potentiates Chondrocyte Anabolism. (A–F) PCR Detection of the Expression of MMP13, SOX9, COL2, ACAN, HIF‐1α, DOT1L Genes in Chondrocytes After Different Treatments; (G,H) Western Blot and Corresponding Calculation Results of HIF1α and Histone Methylation After Different Treatments; (I–L) Immunofluorescence Staining and Corresponding Statistical Results of COL2 and HIF1α in Chondrocytes After Different Treatments; (M–O) Gene Expression of IL‐6, COL2, MMP‐13 After Different Condition Medium Treatment; (P) Schematic Diagram of the Condition media Preparation for Chondrocytes Treatment; (Q,R) Immunofluorescence Staining of COL2 and IL‐6 After Different Condition Medium Treatments. Data Are Expressed as Mean ± SD (*n* = 3). *p < 0.05, **p < 0.01, ***p < 0.001.

It is well‐established that physiological hypoxia promotes chondroycte anabolism which is essential for hyaline cartilage formation, whereas normoxia suppresses this process [52, 53, 54]. Thus, maintaining a localized hypoxia microenvironment is crucial for sustaining chondrocyte metabolism homeostasis and supporting subsequent tissue repair. As a photocontrollable system, the encapsulated alga in the R‐alga hydrogel consume oxygen under dark conditions, thereby inducing a localized hypoxic microenvironment. We therefore hypothesized that the R‐alga hydrogel may restore chondrocyte anabolism by eliciting a protective hypoxic response under darkness, which recapitulates the deeply hypoxic environment of the articular cavity. To test this, chondrocytes were treated with the pro‐inflammatory cytokine IL‐1β to induce an OA‐like phenotype, characterized by elevated catabolism and reduced anabolism. The effects of the R‐alga hydrogel on these cells were then evaluated under IL‐1β stimulation and dark conditions. As shown in Figure S14, the fluorescence intensity (red) significantly increased after R‐alga hydrogel treatment, indicating a significant increase in oxygen consumption within chondrocytes due to the respiration effect of alga. We have supplemented the detection of lubricin (PRG4), a key marker of hyaline cartilage function, using immunofluorescence. Treatment with R‐alga hydrogel significantly upregulates lubricin expression compared to the R‐Alg + Red group (with oxygen production), confirming the restoration of hyaline cartilage function (Figure S15). We further utilized PCR and immunofluorescence assays for validation (Figure 4A–F). The results demonstrated that, in comparison to the control group, the IL‐1β group exhibited significantly elevated levels of MMP13, while the levels of SOX9, COL2 and ACAN markedly reduced. These findings suggest pronounced degeneration responses in chondrocytes. Treatment with the hydrogel alone (R group) resulted in only partial improvement. In contrast, the R‐alga group showed significantly enhanced chondrocyte repair capacity, as evidenced by reduced expression of MMP13, and increased expression of SOX9, COL2, ACAN and HIF‐1α. However, the R‐alga+Red group did not sustain the aforementioned reparative effects, with the cells exhibiting an inflammatory phenotype akin to that observed in the IL‐1β group, as evidenced by increased expression of MMP13 and reduced matrix synthesis factor expression. Hence, the oxygen‐consuming activity of cyanobacteria in the dark could generate a localized hypoxic environment, thereby facilitating the accumulation of HIF‐1α and chondrocyte repair potential.

In order to undercover the underlying molecular mechanisms, we focused on histone methyltransferase DOT1L expression, which has been demonstrated as a crucial role in cartilage homeostasis [28]. In comparison to the control group, treatment with IL‐1β alone resulted in a moderate downregulation of H3K79me2 and DOT1L levels. In contrast, the R‐alga group exhibited a significant upregulation of HIF‐1α and its downstream markers, H3K79me2 and DOT1L, suggesting that the cells were subjected to a specific hypoxic microenvironment (Figure 4G,H). Additionally, the immunofluorescence staining (Figure 4I–L) data showed that the COL2 fluorescence signal in the R‐alga group was substantially enhanced, which would be suppressed by red light treatment. Additionally, red light treatment effectively abolished the R‐alga‐induced HIF‐1α expression. This study demonstrates that, under dark conditions, cyanobacteria efficiently create a hypoxic microenvironment by depleting oxygen (R‐alga group). This process activates the HIF‐1α‐DOT1L signaling pathway, which enhances the synthesis of chondrocyte matrix proteins. These findings underscore the importance of a hypoxic microenvironment in facilitating the repair of damaged cartilage.

Following the confirmation of the photocontrollable R‐alga hydrogel's role in dual‐targeted synergistic therapy for OA through differentiated oxygen metabolism reprogramming, respectively. To elucidate macrophage‐chondrocyte crosstalk under Red light irradiation, we prepared and collected three conditioned media, including (#1) control medium, which is composed of conditioned media from naïve macrophages without any treatment, (#2) medium from LPS‐stimulated M1‐polarized macrophages (simulating a pro‐inflammatory OA environment), and (#3) medium from macrophages treated with LPS and R‐alga hydrogel followed by Red light irradiation (i.e., reprogrammed toward an M2‐like phenotype), and used them for further chondrocyte treatment. As illustrated in Figure 4M–P, compared to group #1, #2 conditioned medium significantly inhibited COL2 synthesis in chondrocytes while strongly upregulating the expression of MMP13 and the pro‐inflammatory factor IL‐6, successfully inducing a typical OA‐like chondrocyte phenotype. Crucially, #3 conditioned medium significantly enhancing COL2 production and decreasing the expression of MMP13 and IL‐6 relative to group 2. These results clearly demonstrate that R‐alga + Red, by reprogramming macrophages towards an pro‐regenerative M2 phenotype, effectively reduced the toxic effects of pro‐inflammatory factors secreted by them on chondrocytes, thereby protecting chondrocytes and maintaining their anabolic capacity. Furthermore, immunofluorescence staining of COL2 and IL‐6 in chondrocytes robustly supported these findings (Figure 4Q,R).

Collectively, the R‐alga hydrogel could exert synergistic intervention in key pathological aspects of OA through the photocontrollable oxygen regulation. In the dark, it creates protective hypoxia through cyanobacterial respiration, activating the HIF‐1α‐DOT1L signaling axis to directly promote chondrocyte anabolism and inhibit catabolism. When activated by Red light irradiation, it indirectly improves the cartilage microenvironment by reprogramming macrophages towards the M2 phenotype.

### 2.5 In Vivo Therapeutic Effects Evaluation of R‐alga Hydrogel for Cartilage Degeneration and Synovitis

**FIGURE 5:**
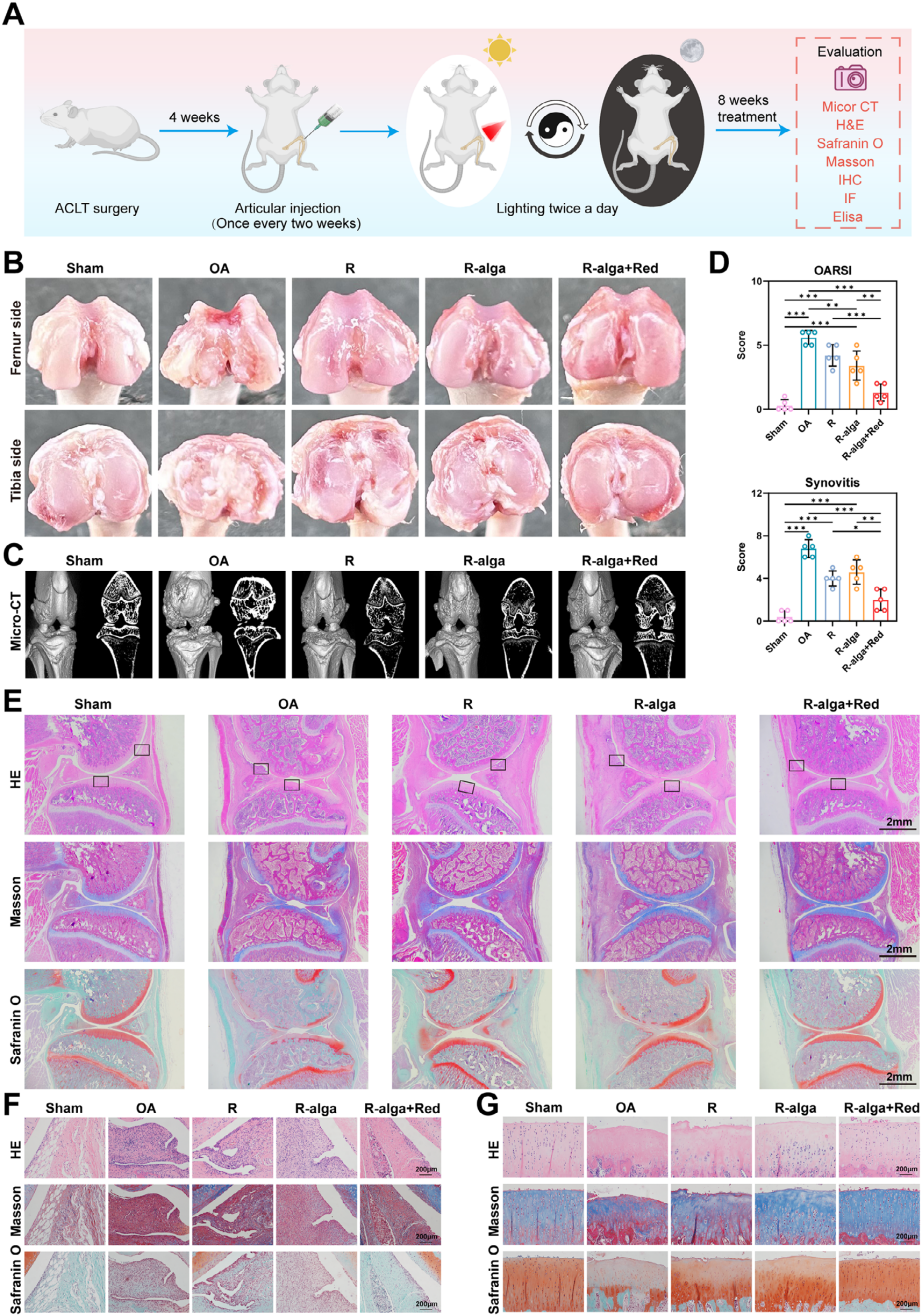
R‐alga hydrogel promotes joint protection and regeneration in OA rats. (A) Schematic diagram of the construction, treatment, and efficacy evaluation of rat knee osteoarthritis; (B) Gross images of knee joints in rats after different treatments; (C) Representative 2D and 3D Micro CT images of knee joints in different groups of rats; (D) Quantification of OARSI scores and Synovitis score for different groups; (E–G) HE, Masson, and Safranin O staining images of knee joint tissues from different groups of rats, as well as enlarged images of local cartilage (F) and synovial tissue (G). Data are expressed as mean ± SD (*n* = 5). *p < 0.05, **p < 0.01, ***p < 0.001.

Inspired by the promising in vitro results, we further systematically evaluated the in vivo therapeutic efficacy of R‐alga hydrogel in an OA rat model induced by Anterior Cruciate Ligament Transection (ACLT). In detail, 4 weeks post‐surgery, treatments were exerted and lasted for 8 weeks via a total of 4 intra‐articular injections as reported (Figure 5A) [55]. To assess the spatial distribution of the hydrogel within the joint, the hydrogel was pre‐labeled with FITC (green), enabling direct visualization of its localization (Figure S16). The images clearly show the hydrogel adhering to the surfaces of both cartilage and synovium. Cyanobacteria were identified by their intrinsic autofluorescence (red). The overlap of green and red signals confirms that the cyanobacteria remained encapsulated within the hydrogel matrix at the target tissue sites. This histological analyses provide definitive evidence of the hydrogel's spatial distribution and its capacity to deliver cyanobacteria to specific joint tissues. The long‐term biosafety and immunogenicity of living cyanobacteria are paramount for clinical translation. Actually, the application of cyanobacteria in biomedical filed has been wildly explored. For instance, researches have found the richness in bioactive substance of cyanobacteria extract, which could enhance superoxide dismutase (SOD) and catalase (CAT) activity in animal study [56]. In another study, researchers injected live cyanobacteria into ischemic hearts, where cyanobacteria's photosynthetic activity directly generated oxygen to rescue hypoxic myocardium [57]. Furthermore, the majority of current epidemiological studies on cyanobacteria suggest that they are harmless to animals [58, 59, 60]. In our study, we also studied the biosafety and immunogenicity of living cyanobacteria through both quantitative cytokine analyses and histological evaluation (Figure S17a–d). Our data demonstrate that at the end of the observation period (day 28), the levels of key pro‑inflammatory cytokines (IL‐1β, TNF‐α, and IL‐6) in the R‐alga + Red group were comparable to those in the control group. H&E staining of the joint tissues demonstrated an absence of inflammatory cell infiltration, synovial hyperplasia, and tissue necrosis in the treatment group. Additionally, peripheral blood samples were collected for comprehensive hematological and biochemical assays, as shown in Figure S17e–l. All tested parameters, including red blood cell count (RBC), hemoglobin (HGB), platelet count (PLT), alanine aminotransferase (ALT), aspartate aminotransferase (AST), lactate dehydrogenase (LDH), and albumin (ALB) were found to be within the normal reference ranges [30]. This suggests that the R‐alga system does not trigger a systemic immune response or chronic inflammation within the joint cavity.

As shown in Figure 5B, the OA group exhibited severe cartilage defects, rough surfaces, and synovial hyperplasia. Although R hydrogel and R‐alga hydrogel treatments would have certain therapeutic effect, the R‐alga + Red treatment displayed the most significant restorative effects. Furthermore, micro‐CT reconstructions (Figure 5C) revealed significantly smoother and more intact joint surfaces, along with a significant reduction in osteophyte numbers and microscopic fissures. Histological staining (HE, Masson and Safranin O/Fast Green) further corroborated these findings, revealing improved cartilage surface continuity, increased chondrocyte numbers, improved deposition of collagen and proteoglycans in the matrix, and reduced synovial inflammatory infiltration after R‐alga + Red treatment (Figure 5E–G). As expected, the Osteoarthritis Research Society International (OARSI) score and Synovitis score were significantly lower in R‐alga + Red compared to the rest (Figure 5D), confirming the enhanced joint protection and regeneration.

**FIGURE 6:**
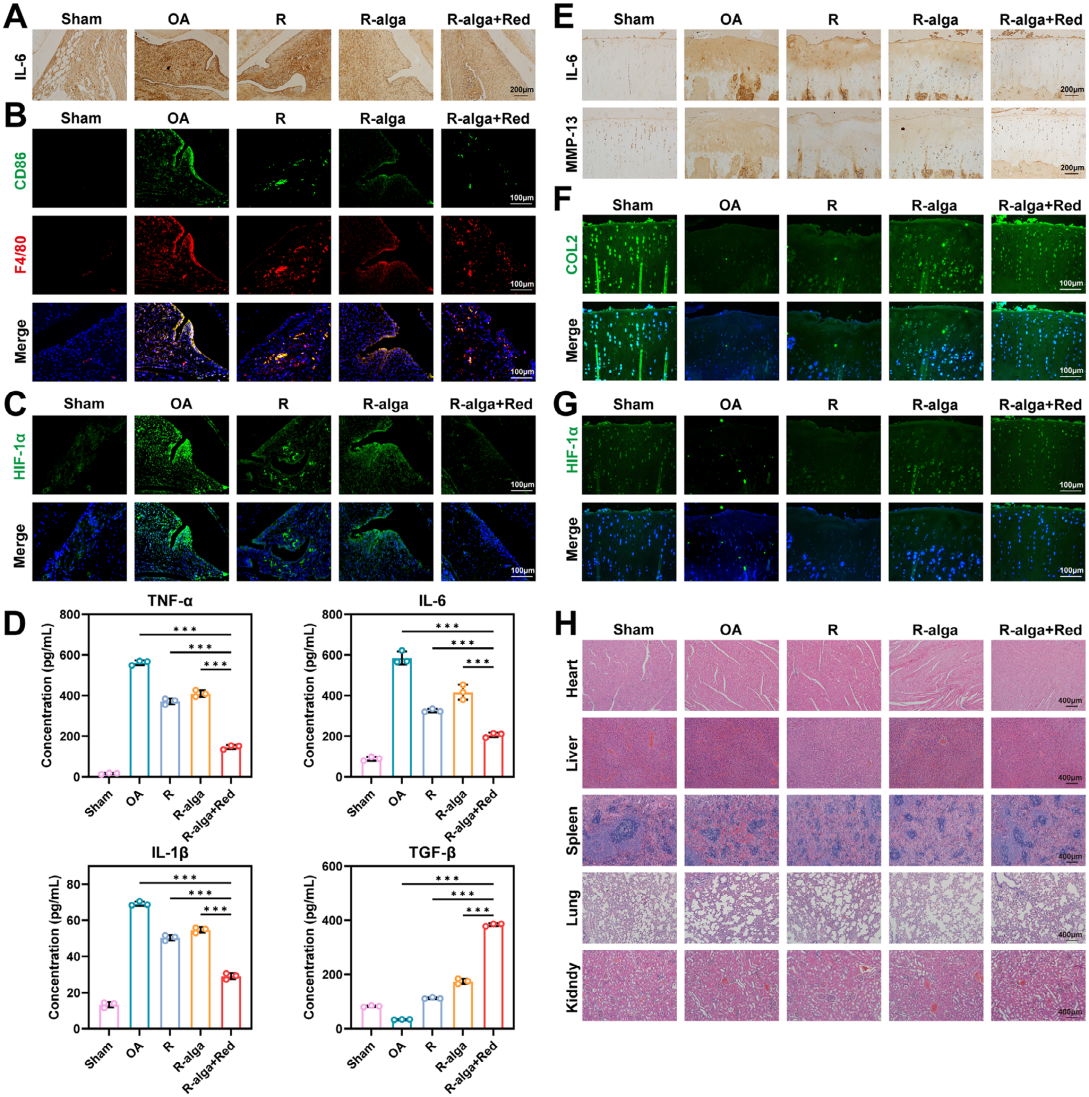
R‐alga hydrogel improves synovial tissue and cartilage microenvironment in osteoarthritic rats. (A) Immunohistochemistry staining of IL‐6 in knee synovium of rats after different treatments; (B, C) Immunofluorescence staining of CD86 and HIF1α in the synovial membrane after different treatments; (D) ELISA analyses for TNF‐α, IL‐6, IL‐1β, and TGF‐β in knee joint fluid of rats after different treatments; (E) Immunohistochemistry staining of IL‐6 and MMP13 in knee cartilage of rats after different treatments; (F) COL2 and (G) HIF1α immunofluorescence staining of knee cartilage after different treatments; (H) HE staining images of different organs after various treatments. Data are expressed as mean ± SD. *p < 0.05, **p < 0.01, ***p < 0.001.

Furthermore, synovial tissue and cartilage samples were harvested following various treatments for comprehensive characterization at cellular and molecular level. First, immunohistochemical and immunofluorescence analyses of synovial sections demonstrated an effective promotion of macrophage repolarization from the pro‐inflammatory M1‐like state (CD86^+^) to the pro‐regenerative M2‐like phenotype, accompanied by downregulation of HIF‐1α and IL‐6 expression, thereby corroborating our in vitro observation (Figure 6A–C). The significant difference among different groups could be further verified by the quantitative analysis (Figure S18). ELISA analysis further confirmed significantly reduced levels of pro‐inflammatory factors (TNF‐α, IL‐6, IL‐1β) and elevated expression of anti‐inflammatory factors (i.e., TGF‐β) in the synovial fluid after R‐alga + Red treatment compared to the rest groups (Figure 6D). Notably, immunohistochemical and immunofluorescence evaluations of cartilage tissues showed significant inhibition of IL‐6 and MMP‐13 expression, alongside upregulated expression of the anabolic marker COL2 and HIF‐1α after R‐alga + Red treatment compared to the rest group (Figure 6E–G and Figure S19). Meanwhile, HE staining of major organs revealed no significant pathological abnormalities (Figure 6H), supporting the good biocompatibility of the as‐prepared hydrogel.

Collectively, the photocontrollable R‐alga hydrogel establishes a smart intra‐articular oxygen dynamic balance system. Under Red light irradiation, it could locally release oxygen to ameliorate hypoxia, suppresses HIF‐1α expression, and drives macrophage M2‐like repolarization, thereby alleviating synovitis and systemic inflammation. Moreover, it consumes oxygen to maintain physiological hypoxia under dark, activating HIF‐1α and its downstream protective pathways to directly promote cartilage anabolism and repair. Through these dual targeting mechanisms, the R‐alga synergistically and precisely mitigates cartilage degeneration and synovitis, ultimately promoting the functional recovery of OA joint tissues.

### 2.6 Multi‐Omics Elucidates the Crosstalk and Regulatory Molecular Networks

**FIGURE 7:**
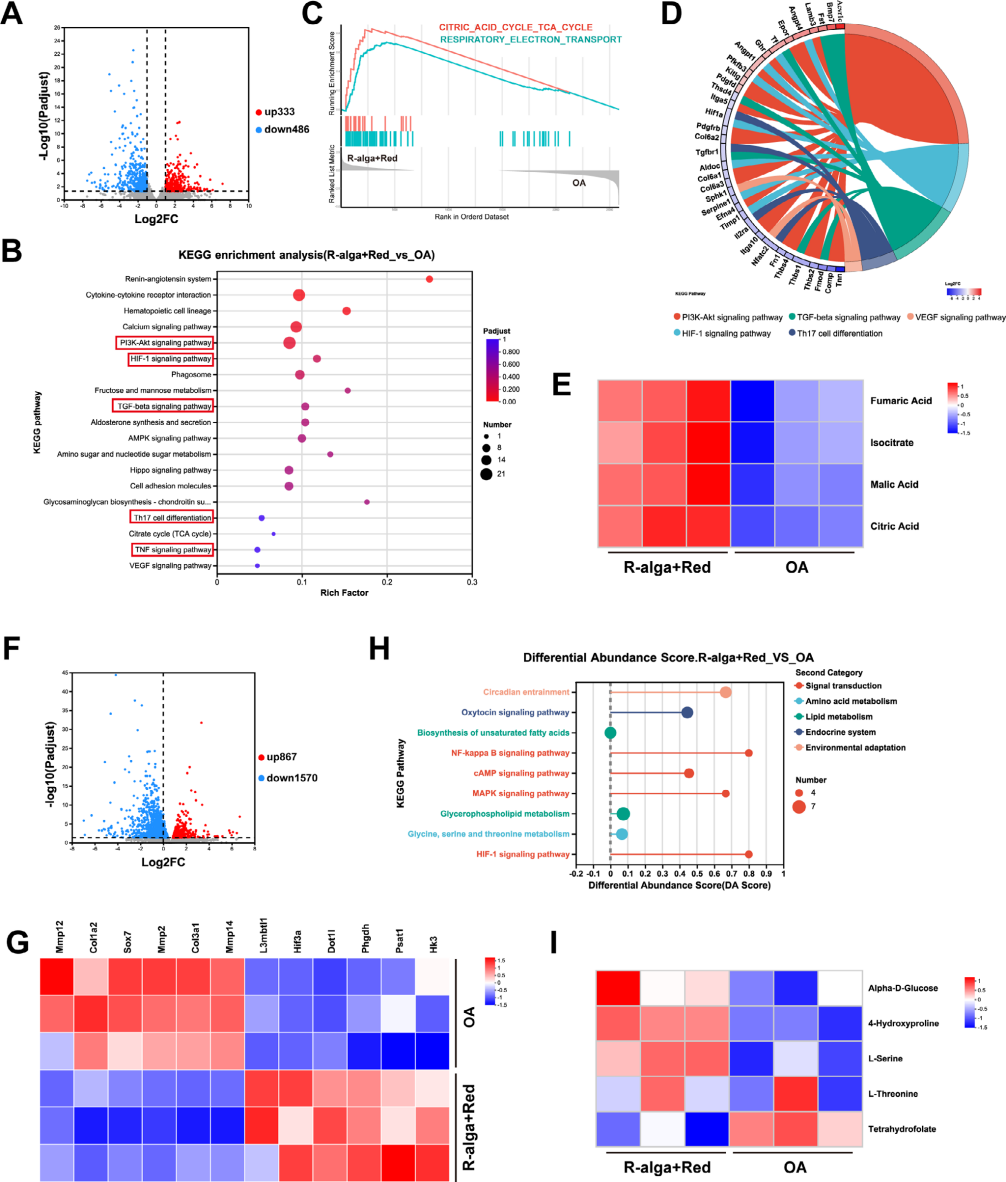
Multi‐Omics elucidates the crosstalk and regulatory molecular networks underlying hydrogel‐mediated efficient therapeutic effects. (A) Volcanic map of DEGs in the transcriptome of joint synovial membrane; (B) synovial transcriptome KEGG gene enrichment analyses; (C) synovial transcriptome GSEA gene enrichment analyses; (D) KEGG enrichment string plot of synovial transcriptome; (E) Expression of TCA related metabolites in the synovial metabolome; (F) Volcanic map of DEGs in the transcriptome of joint cartilage; (G) Gene clustering analysis of DEGs in joint cartilage transcriptome; (H) KEGG enrichment analyses of the joint cartilage metabolome; (I) DEGs clustering analysis of serine metabolites in the joint cartilage metabolome.

Due to the excellent therapeutic effects of R‐alga hydrogel, we further performed RNA sequencing and Metabolomic analyses on synovial and cartilage tissues to systematically elucidate the underlying crosstalk and regulatory molecular networks. RNA sequencing analysis of synovial tissue identified 819 differentially expressed genes (DEGs), comprising 333 upregulated and 486 downregulated genes (Figure 7A). Kyoto Encyclopedia of Genes and Genomes (KEGG) pathway enrichment analysis showed that these DEGs were significantly enriched in the PI3K‐AKT signaling pathway, HIF‐1α signaling pathway, TGF‐β signaling pathway, Th17 cell differentiation, and VEGF signaling pathway [61, 62, 63] (Figure 7B,D), suggesting that the R‐alga + Red treatment modulates hub networks governing inflammation, immune response, hypoxia adaption, and agiogenesis. More interestingly, respiratory chain electron transport and tricarboxylic acid (TCA) cycle‐related pathways [64] (Figure 7C) were also significantly enriched in Gene Set Enrichment Analysis (GSEA), indicating that photocatalytic oxygen generation by R‐alga + Red mitigates synovial hypoxia and promotes energy metabolism recovery. Additionally, metabolomic profiling of synovial tissue identified 489 differential metabolites compared to the OA group, the R‐alga + Red treatment significantly increased the levels of key TCA cycle intermediates (e.g., citrate, isocitrate, malate, fumarate) [65] (Figure 7E). These results strongly support the restoration of synovial tissue aerobic metabolism and enhanced mitochondrial function, thereby promoting HIF‐1α degradation and inhibiting of downstream inflammatory cascade in synovial tissue after treatment.

In cartilage, transcriptomic profiling revealed a broader changes after R‐alga treatment, identifying 2437 DEGs, including 867 upregulated and 1570 downregulated genes. KEGG analysis showed DEGs significantly enriched in extracellular matrix (ECM) organization and collagen‐mediated ECM interaction‐related pathways (Figure S20), consistent with the observed histological improvements in repaired cartilage (Figures 5 and 6). More importantly, R‐alga treatment significantly downregulated multiple catabolic markers [66, 67] associated with cartilage degradation, such as MMP12, MMP2, MMP14, Col1a2 and Col3a1 (Figure 7F,G). Conversely, the gene expression of key cartilage protective markers upregulated, including hypoxia‐regulating factor HIF3a, histone methyltransferase DOT1L, and genes related to L‐serine biosynthesis [68, 69, 70] (e.g., PHGDH, PSAT1). Further metabolomics analysis identified 629 differential metabolites and revealed significant enrichment in the HIF‐1α signaling pathway, NF‐κB signaling pathway, and serine metabolism pathway (Figure 7H). Meanwhile, metabolites associated with serine metabolism were specially upregulated (Figure 7I). Moreover, immunohistochemistry staining further confirmed a marked increased expression level of serine metabolism markers (Phgdh, Psat1) and histone methylation (H3k79me2) in cartilage tissue of treated rat compared to the OA group (Figure S21). These multi‐level data collectively support that in the dark deep cartilage, the physiological hypoxic environment maintained by cyanobacterial respiration within R‐alga hydrogel continuously activates HIF‐1α and its isoform HIF‐3α signaling. This activation simultaneously suppresses catabolic process (e.g., downregulation of MMPs) and promotes anabolic capacity via DOT1L‐mediated epigenetic regulation and enhanced L‐serine biosynthesis in chondrocytes, potentially affecting methylation donors availability, thereby synergistically promoting ECM repair and regeneration.

Collectively, integrating transcriptomic and metabolomic evidence from synovial and cartilage tissues, this study elucidates the dynamic oxygen regulation mechanism of R‐alga hydrogel at the molecular level. In the photo‐accessible synovial region, oxygen generated via photosynthesis alleviated hypoxia milieu, effectively promoted macrophage repolarization toward the M2 phenotype, and reduced inflammation by activating the TCA cycle, inhibiting HIF‐1α, and modulating key signaling pathways, such as PI3K‐AKT and TGF‐β signaling pathways. In contrast, within the avascular cartilage region, respiration‐driven oxygen consumption maintained and enhanced physiological hypoxia, stabilized HIF‐1α/HIF‐3α, upregulated DOT1L, and promoted serine metabolism. These processes, synergistically activated protective anabolic programs and inhibited catabolism, ultimately achieving precise restoration of the osteoarthritic joint tissue microenvironment.

## 3 Conclusions

In summary, a photocontrollable cyanobacteria‐based hydrogel (R‐alga) that enables precise regulation of intra‐articular oxygen dynamics in OA through the photosynthesis and respiration of cyanobacteria was constructed. Under Red light irradiation, cyanobacteria in the synovial region produce oxygen, which alleviates local hypoxia, inhibits the HIF‐1α signaling pathway, and induces macrophage repolarization toward the pro‐regenerative M2‐like phenotype, thereby significantly mitigating synovitis. In contrast, in the deep cartilage, cyanobacterial respiration consumes oxygen, which activates the HIF‐1α‐DOT1L axis to promote collagen/proteoglycan synthesis while inhibiting the expression of catabolic enzymes in rat chondrocytes. In a rat model of OA, R‐alga combined with Red light treatment resulted in an over 70% reduction in OARSI scores, over 65% reduction in synovial inflammatory infiltration, and effective restoration of cartilage matrix structure and composition. Multi‐omics analysis further demonstrates that photosynthesis in the synovial region enhanced anti‐inflammatory effects by activating PI3K‐AKT/TGF‐β pathways and restoring the TCA cycle. Conversely, in the cartilage region, hypoxia‐dependent DOT1L epigenetic regulation and serine metabolism reprogramming synergistically promoted anabolism. Moreover, the R‐alga hydrogel also exhibits excellent injectability, self‐healing properties, and biocompatibility. Collectively, this study overcomes the limitations of traditional inert biomaterials in functional regulation, pioneering the microorganism oxygen metabolism engineering therapeutic strategy, achieving dynamic regulation and synergistic repair of the osteoarthritic microenvironment, and opening a new path for the construction of intelligent OA treatment systems.

Despite these significant advances, certain limitations must be addressed to facilitate clinical translation. First, regarding light penetration, the efficacy of 630 nm LED irradiation may be compromised in human joints with synovial thickening or effusion, warranting further validation in large animal models. Second, although favorable biocompatibility has been observed in rat studies, the long‐term fate of cyanobacteria and potential immune responses in humans remain to be established in clinical trials.

## 4 Experimental Section

### 4.1 Materials

Hyaluronic acid (HA), 3‐aminophenylboronic acid (PBA), and dimethyl sulfoxide (DMSO) were purchased from Sigma‐Aldrich (China). Adipic dihydrazide (ADH), 1‐(3‐dimethylaminopropyl)‐3‐ethylcarbodiimide hydrochloride (EDC), and N‐hydroxysuccinimide (NHS) were obtained from Macklin (China). Fetal bovine serum (FBS) and penicillin/streptomycin were purchased from Procell (China). FITC anti‐mouse CD86 antibody and PE anti‐mouse CD11b antibody were obtained from BD Biosciences (USA). The CCK‐8 assay kit, Hoechst, and dialysis bags were purchased from Solarbio (China).

### 4.2 Cyanobacteria Culture

At room temperature, cyanobacteria (Synechococcus elongatus PCC 7942, collected from the Freshwater Algae Culture Collection of the Institute of Hydrobiology) were cultured in 200 mL BG‐11 medium (blue‐green medium) and stirred occasionally to enhance CO_2_ dissolution [30]. The cultured alga in medium was illuminated with a white light to facilitate regular cyanobacterial photosynthesis.

### 4.3 Synthesis of HA‐ADH‐PBA and Ox‐Dex

HA‐ADH‐PBA was synthesized via an amidation reaction using EDC/NHS [71]. Briefly, HA (1 g, 0.01 mmol) was dissolved in 2 × PBS, and ADH was added. Then, EDC (3.9 g, 0.02 mol) and NHS (1.15 g, 0.01 mol) were added. The reaction was continued at 37°C for 12 h (pH = 6). The resulted HA‐ADH was further reacted with PBA to obtain HA‐ADH‐PBA. Ox‐Dex was obtained through oxidation reaction. In detail, dextran (1 g, 0.014 mmol, Mw = 70 kDa) was dissolved in 100 mL of water at 37°C in a water bath, and NaIO_4_ (535 mg, 2.47 mmol) was added under light protection. After 8 h, 140 µL of ethylene glycol was added to stop the reaction. The mixture was dialyzed (MWCO = 3500 Da) for 5 days and freeze‐dried to obtain Ox‐Dex. The structures of the above polymers were analyzed using a 1H nuclear magnetic resonance (1H NMR) spectrometer (Bruker AVANCE III 400 M, Switzerland).

### 4.4 Preparation of Hydrogels

50 mg of HA‐ADH‐PBA and 100 mg of Ox‐Dex were mixed in 1 mL of PBS containing cyanobacteria (1 × 10^7^ cells/mL) to form R‐alga hydrogel. Meanwhile, R hydrogel was prepared with the same formula without cyanobacteria addition, and half of HA‐ADH‐PBA (25 mg) and Ox‐Dex (50 mg) were employed for Low‐R hydrogel construction.

### 4.5 Physical and Chemical Characterization of Hydrogels

The chemical properties of hydrogels were characterized by Fourier transform infrared spectroscopy (FTIR, Nicolet 6700, US). R and R‐alga hydrogels with different concentrations after complete crosslinking were freeze dried and observed by scanning electron microscope (SEM, TESCAN VEGA3LMU, Czech Republic). Moreover, the rheological properties of hydrogels were tested by a rotational rheometer (HAKE, RS6000, US), including time sweep, strain sweep, complex viscosity frequency sweep, and high and low strain cyclic sweep. Furthermore, the compressive mechanical properties were evaluated by quasi‐static compression tests using a universal material testing machine (Instron 3400, US). For degradation testing, the hydrogels were incubated at 37°C in three different degradation media, namely PBS, acidic solution with pH = 5 and 5 mM H_2_O_2_ solutions, respectively. At the planned time points, the hydrogel samples were freeze‐dried and weighed.

### 4.6 Free Radical Scavenging Activity Evaluation

DPPH method was employed for testing the free radical scavenging activity of as‐prepared hydrogel. In brief [72, 73], DPPH was dissolved in a mixture of ethanol and water (v:v = 1:1) to a concentration of 200 µM. Then, 1 mL of the DPPH solution was added to a 2 mL tube containing 50 µL of hydrogel, and the mixture was further kept at 37°C. At 0.5, 3, 6, 9, and 15 h, 150 µL of the DPPH solution was transferred to a 96‐well plate, and the absorbance at 517 nm was measured using a microplate reader. The scavenging efficiency was calculated as % inhibition = (AB—as)/(AB—AK), where AB, as, and AK represent the absorbance of the control (only DPPH), sample (DPPH + hydrogel), and blank plate, respectively.

### 4.7 In Vitro Oxygen Production and Consumption Capacities Detection

10 mL of PBS was incubated with cyanobacteria or R‐alga hydrogel at 25°C under illumination with a 630 nm near‐infrared LED light [74]. The dissolved O_2_ concentration was measured at regular intervals using an O_2_ probe (Rex, JPBJ‐605). To examine the oxygen consumption capacity of R‐alga, repeated light cycles were performed. The effect of photosynthesis on O_2_ production and consumption was evaluated by turning on the light for 60 min and off for 120 min. Moreover, the stability of R‐alga was examined through dispersing hydrogel in PBS for 1, 3, 6, 9, 12, and 15 days, and then the release and consumption of O_2_ were monitored.

### 4.8 Cell Culture

RAW 264.7 murine macrophages were purchased from servicebio (Cat. No. STCC20020; RRID: CVCL_0493) and the cell line was authenticated by short tandem repeat (STR) typing before use to ensure cell identity. Primary rat articular chondrocytes were isolated from the articular cartilage of 3–7 days‐old male Sprague‐Dawley (SD) rats. The isolation and culture procedures were conducted in our laboratory in accordance with standard protocols established in the previous literature [75]. Both cell types were cultured at 37°C under 5% CO_2_ in high‐glucose Dulbecco's Modified Eagle Medium (DMEM; Gibco, USA) supplemented with heat‐inactivated fetal bovine serum (10% FBS), streptomycin (100 mg mL^−1^), and penicillin (100 mg mL^−1^).

### 4.9 Intracellular ROS Detection

Intracellular ROS generation was detected using the DCFH‐DA (ROS fluorescent probe), which could be oxidized to generate highly fluorescent DCF by free radicals. Rat chondrocytes (1×10^5^ cells/well) were cultured in 6‐well plates for 24 h, and then treated with different methods (blank, H_2_O_2_, H_2_O_2_+R, H_2_O_2_+R‐alga, H_2_O_2_+R‐alga + Red) for 4 h. H_2_O_2_+R‐alga + Red group cells were exposed to 630 nm LED red light (irradiation intensity: 50 mW/cm^2^) for 20 min. After that, these cells were incubated with DCFH‐DA (20 µM) for 30 min. Finally, the cells were washed three times with PBS, analyzed under a fluorescence microscope, and detected by flow cytometry.

### 4.10 Induction of Arthritic Chondrocytes

Rat chondrocytes at the logarithmic growth stage were seeded in 6‐well plates at a density of 1×10^5^ cells/well, and then stimulated with IL‐1β (5 ng/mL) for 24 h to induce arthritic state. After that, they were divided into four groups: model group (IL‐1β, 5 ng/mL), R group, R‐alga group, and R‐alga+Red group. Meanwhile, chondrocytes without IL‐1β treatment were used as control group. These cells were further treated for 12 h and collected for subsequent experiments.

### 4.11 Macrophage Polarization

M0 macrophages at the logarithmic growth stage were seeded in 12‐well plates at a density of 2 × 10^5^ cells/well. After overnight culture, adherent cells were selected and stimulated with lipopolysaccharide (LPS, 20 ng mL^−1^) for 24 h to obtain M1‐like macrophages. After that, M1‐like macrophages were divided into 4 groups, including Control group, R group, R‐alga group and R‐alga+ Red group. Meanwhile, M0 macrophages were employed as blank group without any treatment. These cells were treated for 12 h and collected for subsequent experiments.

### 4.12 In Vitro ATP Evaluation

To assess the energy metabolites quantitatively [76], polarized M1 and M2 RAW 264.7 macrophages were seeded in 6‐well plates at a density of 1 × 10^6^ cells/well and received different treatments (con, LPS, LPS+R, LPS+R‐alga, LPS+R‐alga+Red) for 12 h. After that, cells were washed with PBS and harvested for analysis. ATP was quantified using an ATP detection kit (S 0026, Beyotime) according to the manufacturer's instructions.

### 4.13 In Vitro Oxygenation Detection

The intracellular oxygen level in rat chondroncytes and RAW246.7 macrophages was detected using a hypoxia‐sensitive Ru(dpp)_3_Cl_2_ fluorescence probe after different treatments. Briefly, the oxygen consumption capacity of R‐alga hydrogel was firstly examined when it was co‐cultured with arthritic chondrocytes (IL‐1β group) under a dark condition. The chondrocytes (1 × 10^5^ cells/well) and hydrogel were cultured in 6‐well plates for 10 h under dark conditions. Then, the oxygen consumption capacity of rat chondrocytes co‐cultured with hydrogel was evaluated.

Oxygen production capacity of R‐alga under 630 nm near‐infrared light was evaluated when it was co‐cultured with RAW 264.7 cells. In brief, mouse macrophages (1× 10^6^ cells/well) were cultured in 6‐well plates for 10 h. After cell adhesion, macrophages were placed in an anaerobic culture bags containing commercial O_2_ stripping catalysts and cultured for another 12 h. Then, the medium was replaced with medium containing 100 µL Ru(dpp)3Cl_2_ (500 µg/mL DMSO stock), and the hypoxic condition was maintained for 4 h. Following which, the cells were thoroughly washed with fresh PBS and R‐alga hydrogel containing PCC 7942 (1 × 10^7^ cells/mL) was added for co‐culture. The cells were irradiated with 630 nm Red light for 30 min and then co‐incubated in a closed system for 1 h. Finally, the cells were washed twice and analyzed by fluorescence microscopy.

### 4.14 Immunofluorescence Assays

Rat chondrocytes and RAW 264.7 macrophages were seeded in confocal culture dishes and induced to obtain arthritic chondrocytes and M1‐like macrophages as described above. After different treatments, the cells were washed with PBS and fixed with 4% paraformaldehyde. Then, cells were permeabilized with 0.1% Triton X‐100 for 15 min and blocked with 4% BSA at room temperature for 30 min. Between each step, the cells were washed three times with PBS. After that, cells were immunolabeled with primary antibodies, washed with PBS, and incubated with fluorescent secondary antibodies for 1 h. Nuclei were stained with Hoechst 33342. Finally, cells were analyzed using a confocal fluorescence microscope.

### 4.15 Flow Cytometry Analysis

Cells after different treatments were collected and resuspended in PBS containing 1% BSA. The cell concentration was adjusted to 1 × 10^6^ cells/mL, and 1 × 10^6^ cells were collected for further analysis. Then, cells were resuspended in 100 µL 1% BSA with 1 µL CD86 fluorescent primary antibody and incubated in the dark at 4°C for 30 min. After that, cells were collected by centrifugation and washed with PBS containing 1% BSA. Finally, cells were collected and resuspended in 200 µL PBS containing 1% BSA, and analyzed by flow cytometry. Data analysis was performed using FlowJo software.

### 4.16 Quantitative Real‐Time PCR Analysis

The target gene expression at transcriptive level were quantified by quantitative real‐time polymerase chain reaction (qRT‐PCR) using the primer pairs listed in Table S1. Briefly, RNA was extracted from treated cells according to the protocol of the RNA isolation kit, and cDNA was synthesized using a reverse transcription kit (Fermentas Company, USA). qPCR was performed using a qPCR cycling instrument (Applied Biosystems) under the following conditions: 95°C pre‐denaturation for 3 min, 95°C denaturation for 5 s, 45 cycles, 60°C annealing for 30 s, 45 cycles. GAPDH served as the housekeeping gene.

### 4.17 Western Blot Analysis

Protein extraction and Western blot analysis were carried out as described in the previous study [77, 78]. In brief, total protein was extracted using the radioimmunoprecipitation assay buffer according to the manufacturer's guidelines. 10 µg of protein was separated by sodium dodecyl sulfate polyacrylamide gel electrophoresis and transferred to a polyvinylidene fluoride membrane. After blocking with 5% bovine serum albumin, the membrane was incubated with the primary antibody at 4°C overnight, then washed three times with tris‐buffered saline containing 0.1% Tween 20 (TBST) for 15 min each time. The membrane was then incubated with the secondary antibody at room temperature for 1 h, washed three times with TBST for 15 min each time. The protein blot was visualized using the Western ECL detection kit (Thermo Fisher Scientific, USA) and the Amersham i1 Ultra High Sensitivity Imaging System (UK). Quantification was performed using the digital image analysis software ImageJ. Western blot analysis was performed in triplicate.

### 4.18 In Vivo Osteoarthritis (OA) Rat Model Establishment and Therapeutic Efficacy Evaluation

The animal experiment was approved by the Ethics Committee of Guangxi Medical University (No.202311018). In detail, twelve‐week‐old male SD rats (*n* = 32) were anesthetized with 3% pentobarbital sodium (40 mg/kg) and underwent anterior cruciate ligament transection of the knee joint. After 3 days postoperation, antibiotics (penicillin, intramuscular injection 100,000 units per day) were administered 3 days to prevent postoperative infection and animal licking of the wound. Meanwhile, two exercise training sessions were conducted weekly. One month later, the osteoarthritis model was successfully established. The OA rats were divided into 4 groups (*n* = 8 each), and received different treatments, including PBS, R, R‐alga, and R‐alga+Red, respectively. Of note, the intra‐articular injections would carried out once for every 2 weeks, and the OA rats treated with R‐alga+Red would received Red irradiation twice a day for 20 min. Moreover, another 8 healthy rats without operation would served as control group. Eight weeks after different treatments, fresh knee joint cartilage and synovial tissue were collected for transcriptome and metabolome analyses. Furthermore, the knee joint was fixed in 4% paraformaldehyde, decalcified, and embedded in paraffin for histological evaluation, including hematoxylin and eosin (H&E), Safranin‐O/Fast Green, and immunohistochemical assays. In detail, the collected tissue sections were incubated with rabbit polyclonal anti‐IL‐6, MMP13 (Proteintech, China)) antibodies overnight at 4°C for immunohistochemical staining, then treated with secondary antibody for 1 h, and stained with 3,3'‐diaminobenzidine (DAB) substrate for the paraffin sections. In addition, immunofluorescence staining was also performed, including F4/80, CD86, HIF‐1α, and COL2A1 (Proteintech, China), to evaluate the therapeutic effects. In detail, the corresponding primary antibodies were incubated overnight, and then the sections were treated with FITC or Cy3 Tyramide (Proteintech, China) at 4°C for 30 min, DAPI for 5 min, and then imaged using a fluorescence microscope. Moreover, the enhanced Mankin scoring system was employed to evaluate the pathological state of the knee joint.

### 4.19 RNA Sequencing and Metabolomics Statistics

Knee joint cartilage and synovial tissues of the OA group and the R‐alga+Red group after 8 weeks of treatment were collected. Transcriptome and metabolome analyses were performed to screen for differentially expressed genes (DEGs) and metabolites for further analysis, including principal component analysis (PCA), hierarchical clustering, volcano plot for all significantly DEGs, gene ontology (GO) enrichment analysis, and KEGG pathway analysis.

### 4.20 In Vivo Biological Safety Assessment

Histological observations of the main organs (heart, liver, spleen, lung and kidney) were made using H&E staining to evaluate the biological safety of different treatments.

### 4.21 Statistical Analysis

The data were presented as the mean ± standard deviation (SD). Statistical significance was analyzed using Student's t‐test, one‐way analysis of variance (ANOVA), and Tukey test. When *p < 0.05, **p < 0.01 and ***p < 0.001, it was considered that the differences between groups were statistically significant.

## Author Contributions

**Chong Shen**: methodology, data curation, formal analysis, visualization, investigation, project administration. **Licheng Xi**: investigation, visualization, formal analysis. **Zainen Qin**: conceptualization, writing – review and editing, writing – original draft, resources, supervision, funding acquisition. **Hongjun Huang**: methodology, software, data curation, formal analysis, visualization, investigation, project administration. **Huan Cao**: writing – review and editing, writing – original draft. **Zeyu Huang**: investigation, visualization, formal analysis. **Siyi Liu**: investigation, validation, visualization. **Li Zheng**: conceptualization, supervision, writing – original draft, writing – review and editing, funding acquisition. **Lerong Yang**: methodology, data curation, formal analysis, validation, investigation, visualization, project administration, writing – original draft.

## Conflicts of Interest

The authors declare no conflicts of interest.

## Supporting information




**Supporting File 1**: advs77248‐sup‐0001‐SuppMat.docx.

**Supporting File 2**: advs77248‐sup‐0002‐SuppMat.doc.

**Supporting File 3**: advs77248‐sup‐0003‐SuppMat.pdf.

## Data Availability

The data that support the findings of this study are available from the corresponding author upon reasonable request.
