# Supplementary material for: Engineered Cyanobacteria‐Hydrogel Microecological System for Oxygen Reprogramming Synergizes Immunomodulation With Anabolism in Osteoarthritis Therapy
Source: Adv Sci (Weinh). 2026 Aug 31:e77248. Online ahead of print. doi: 10.1002/advs.77248 (PMC13528180; doi:10.1002/advs.77248)

# Repeat 1

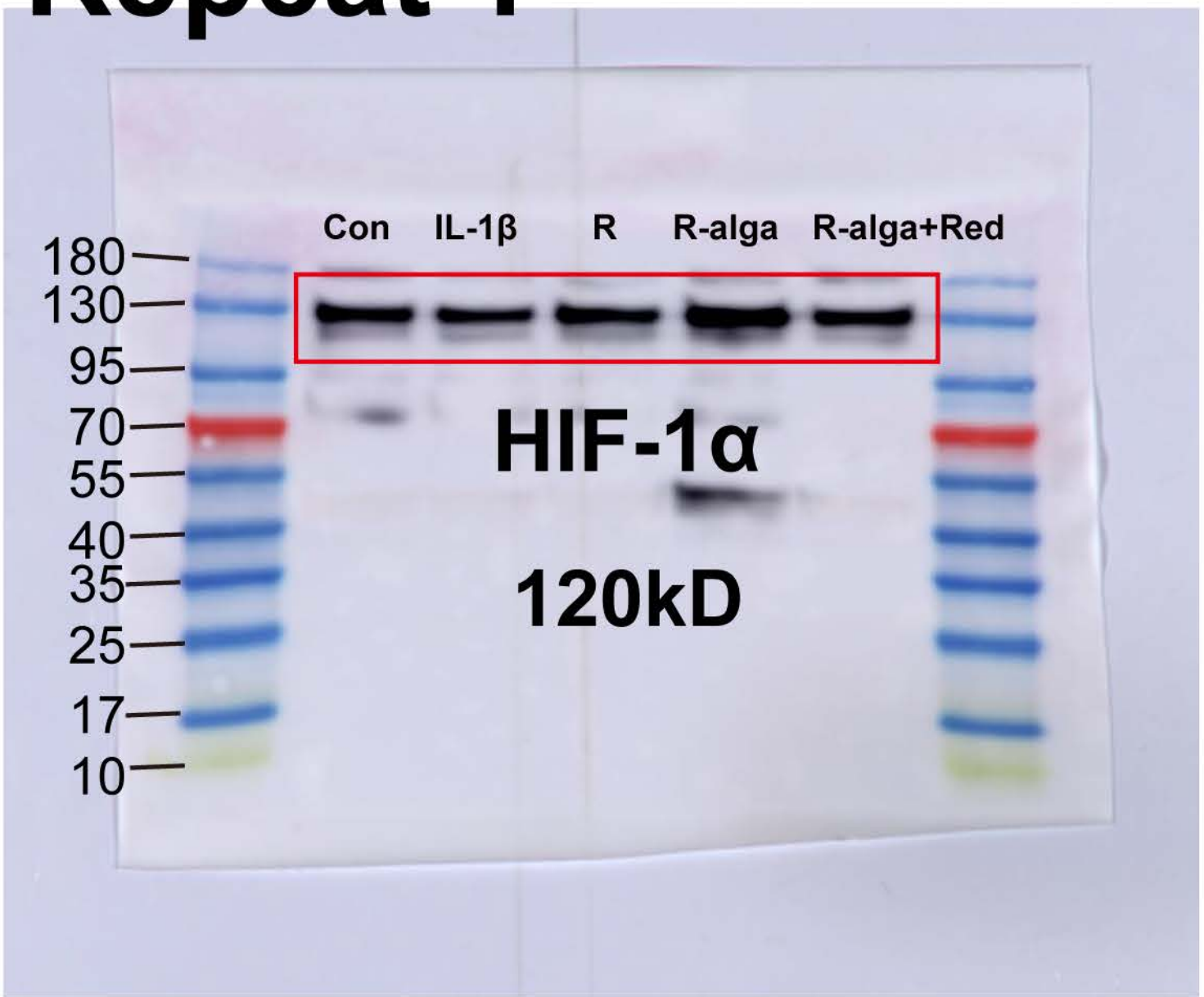


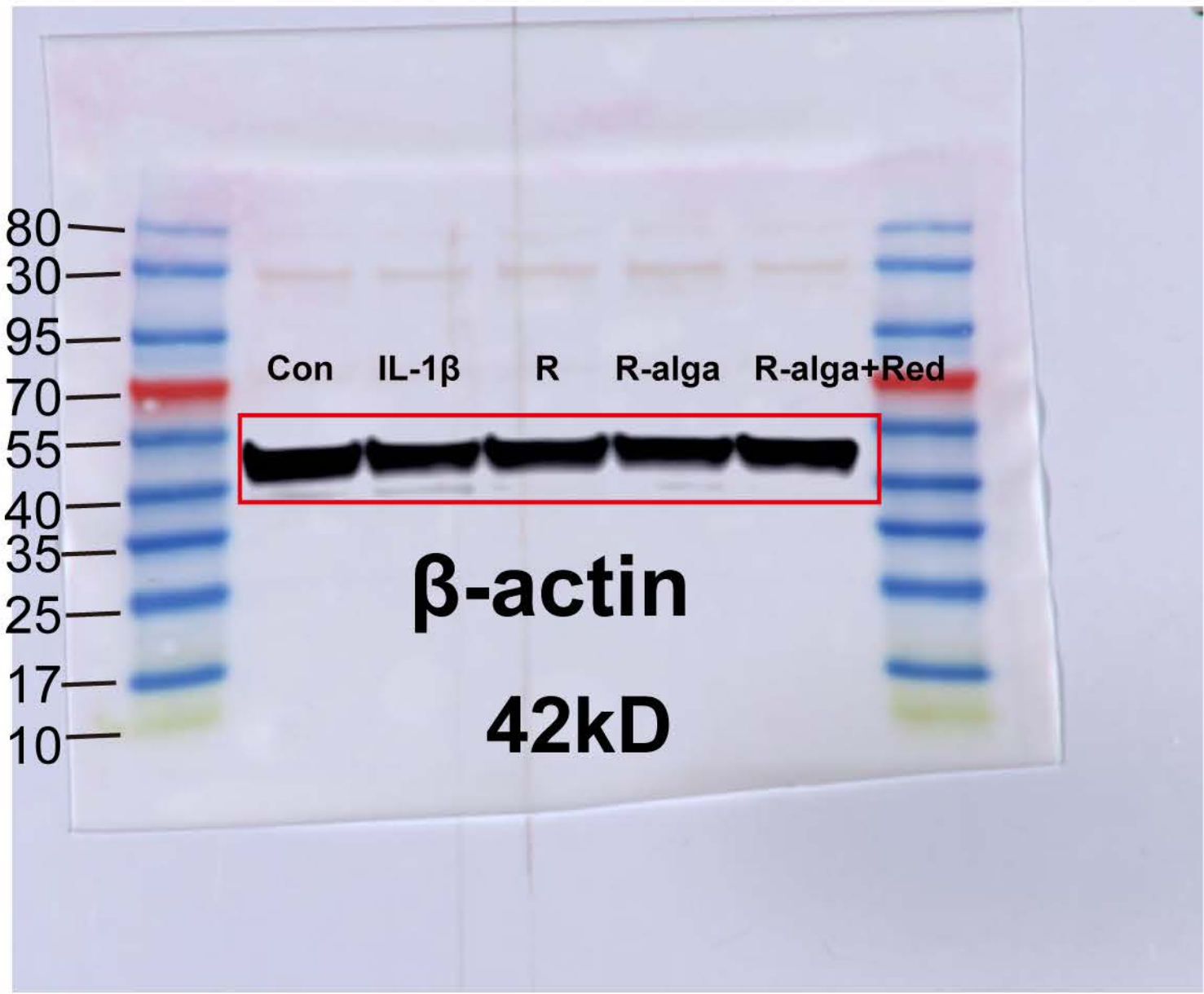


# Repeat 2

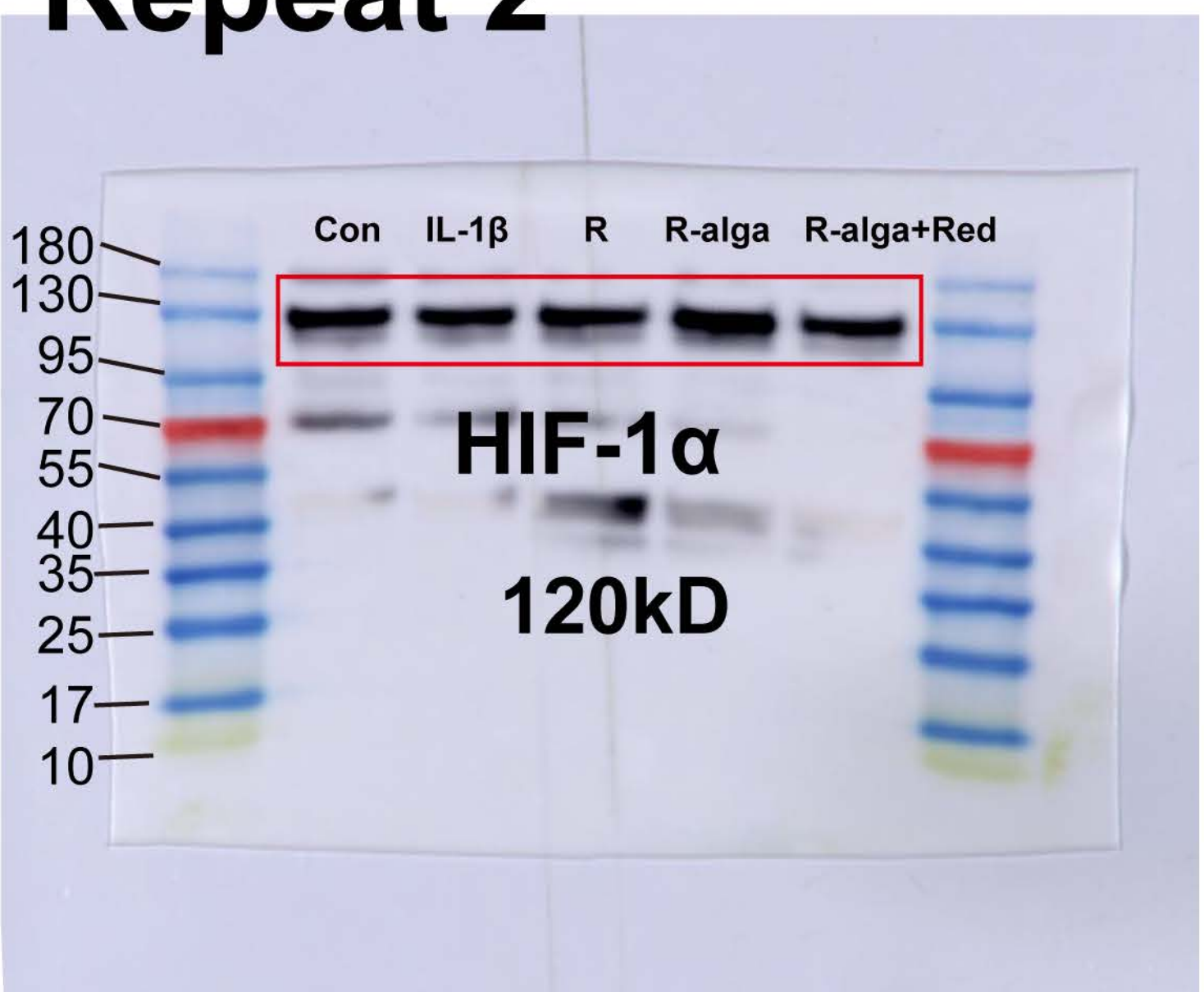


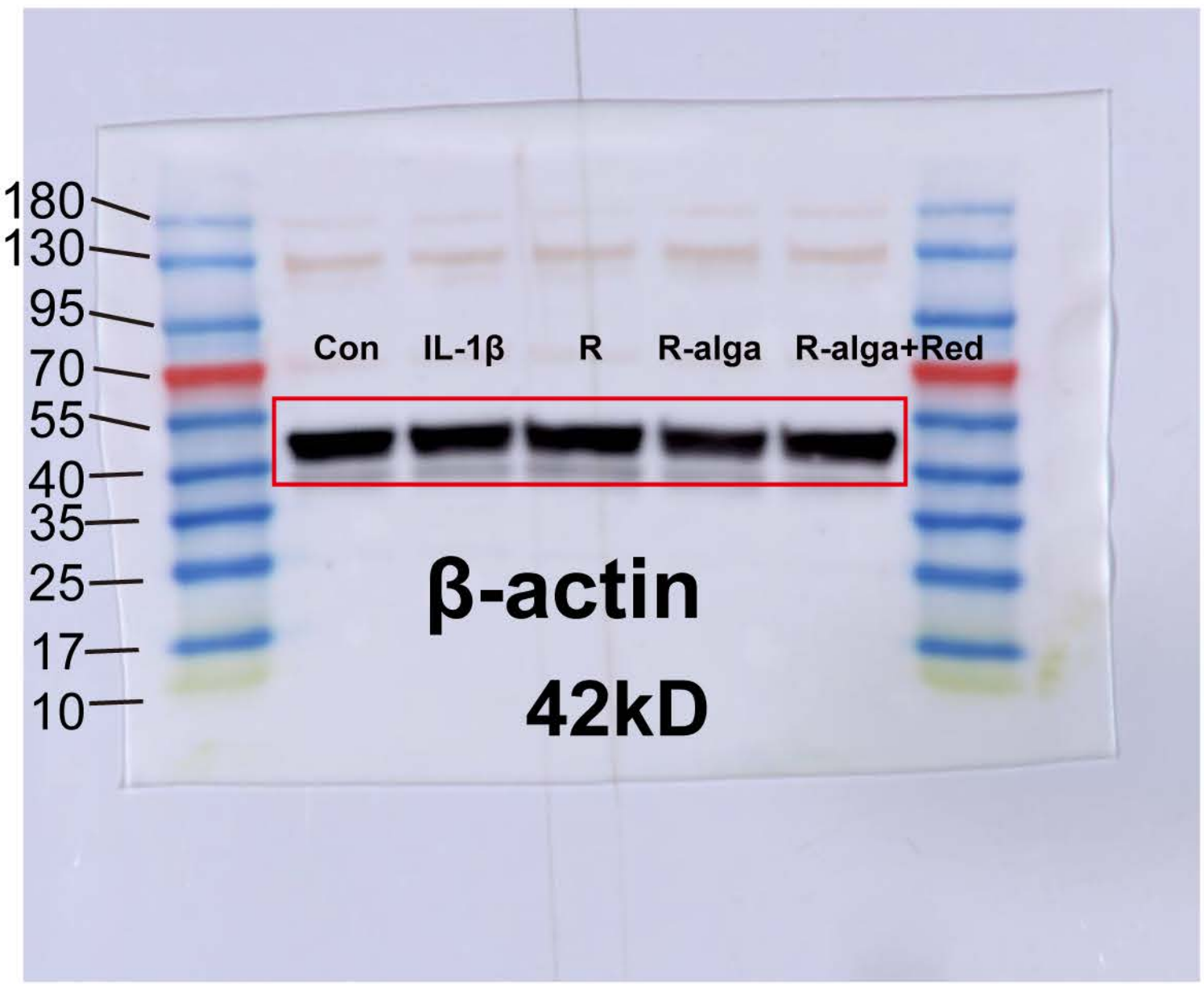


# Repeat 3

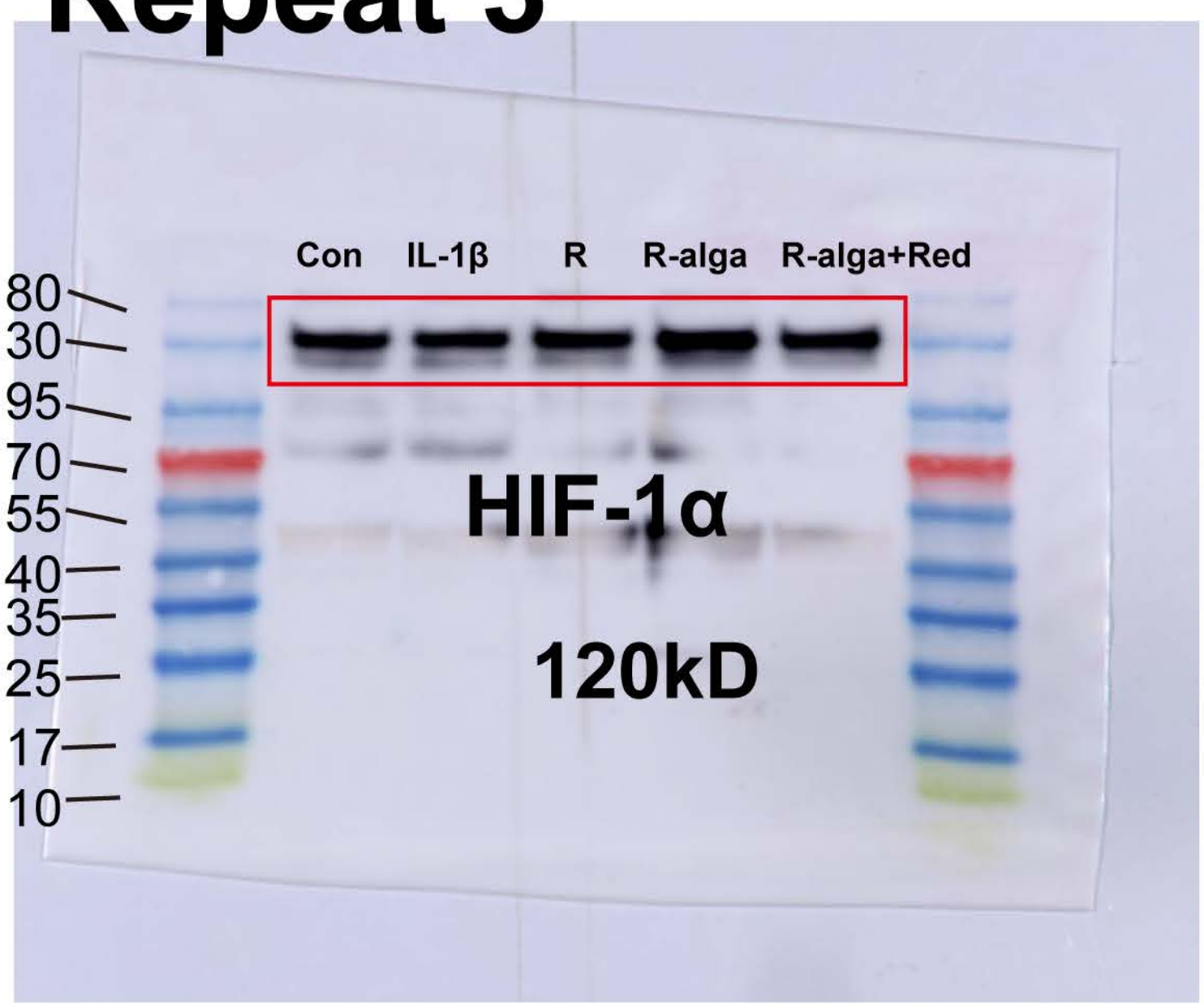


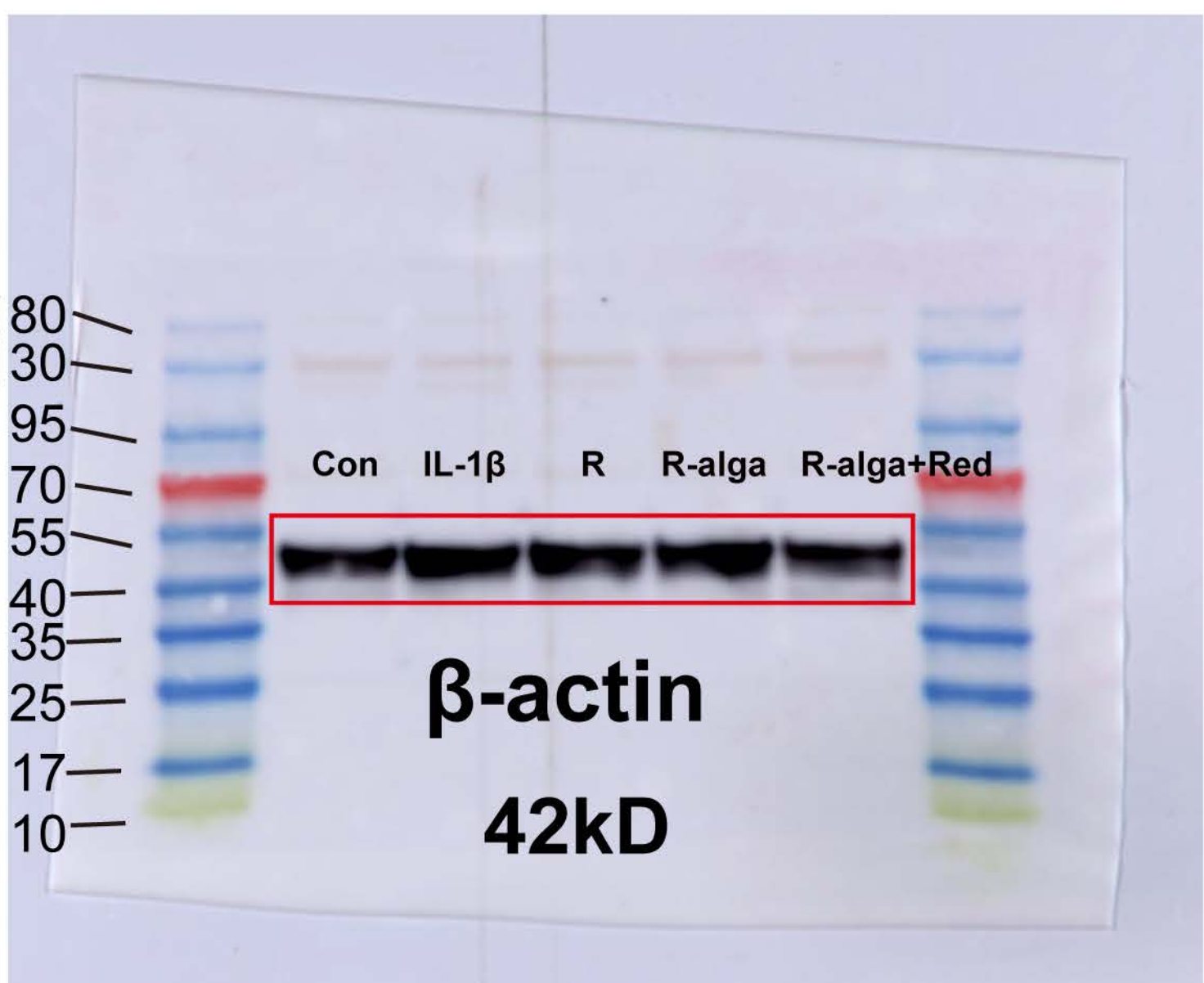

# Repeat 1

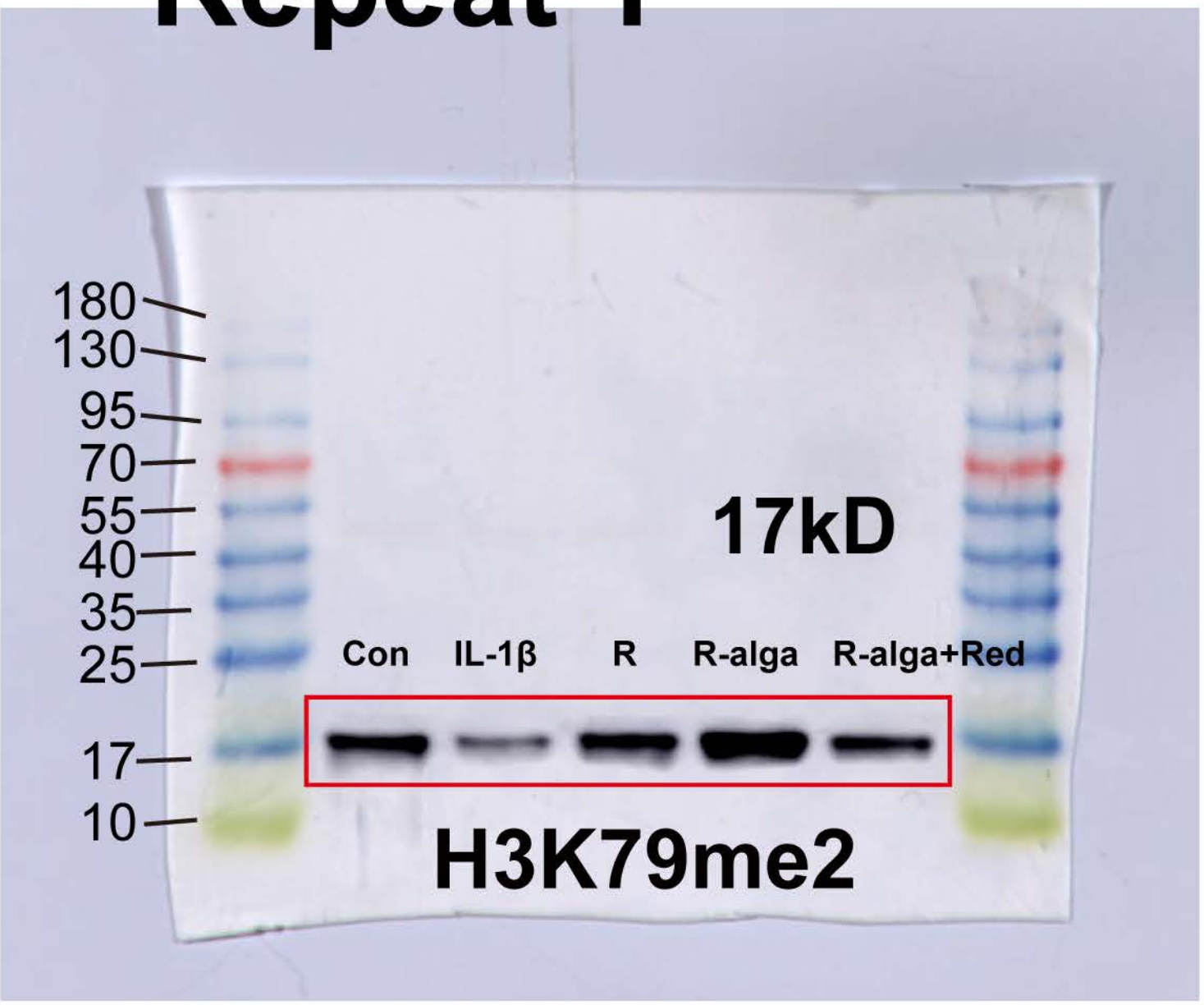


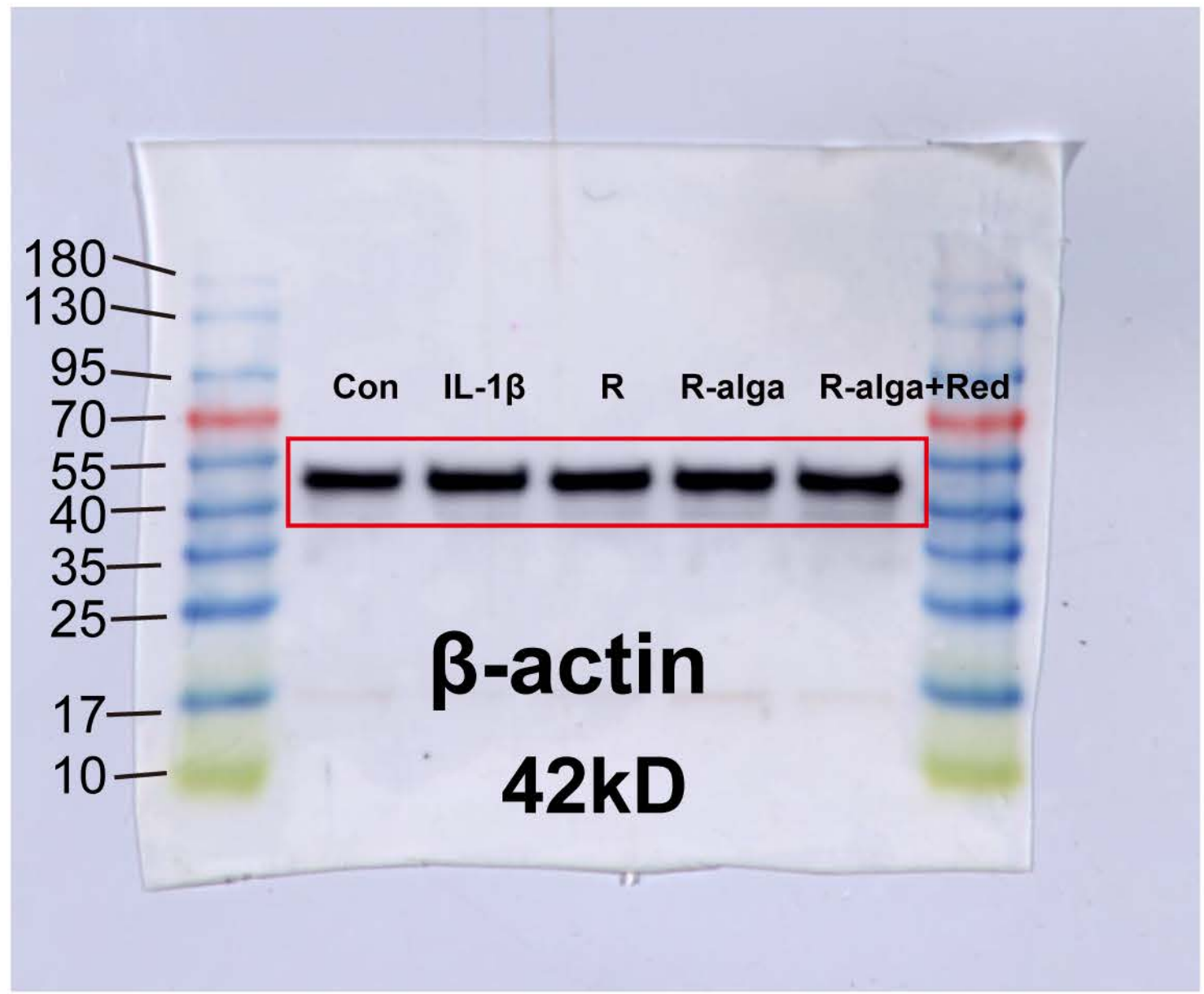


# Repeat 2

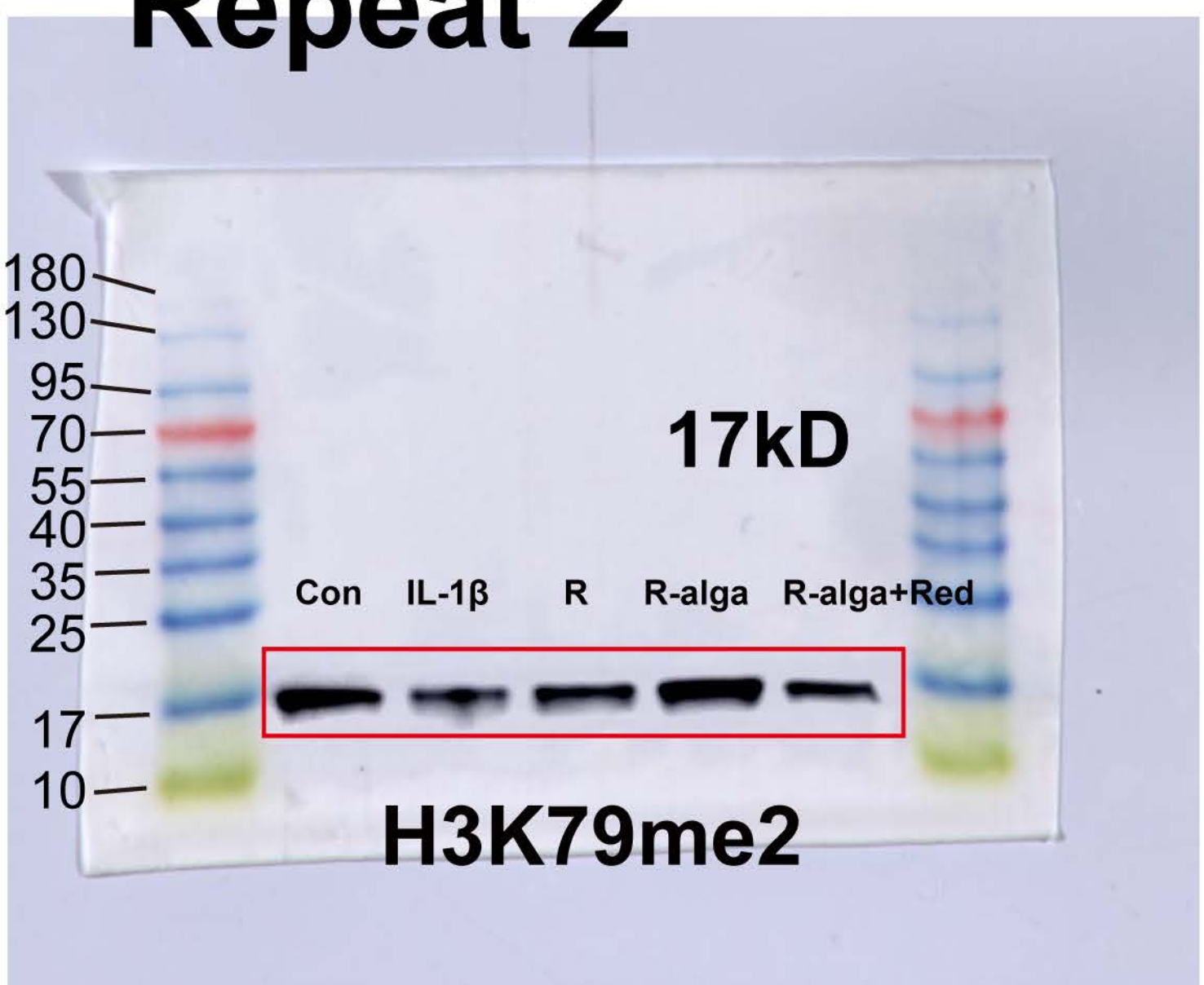


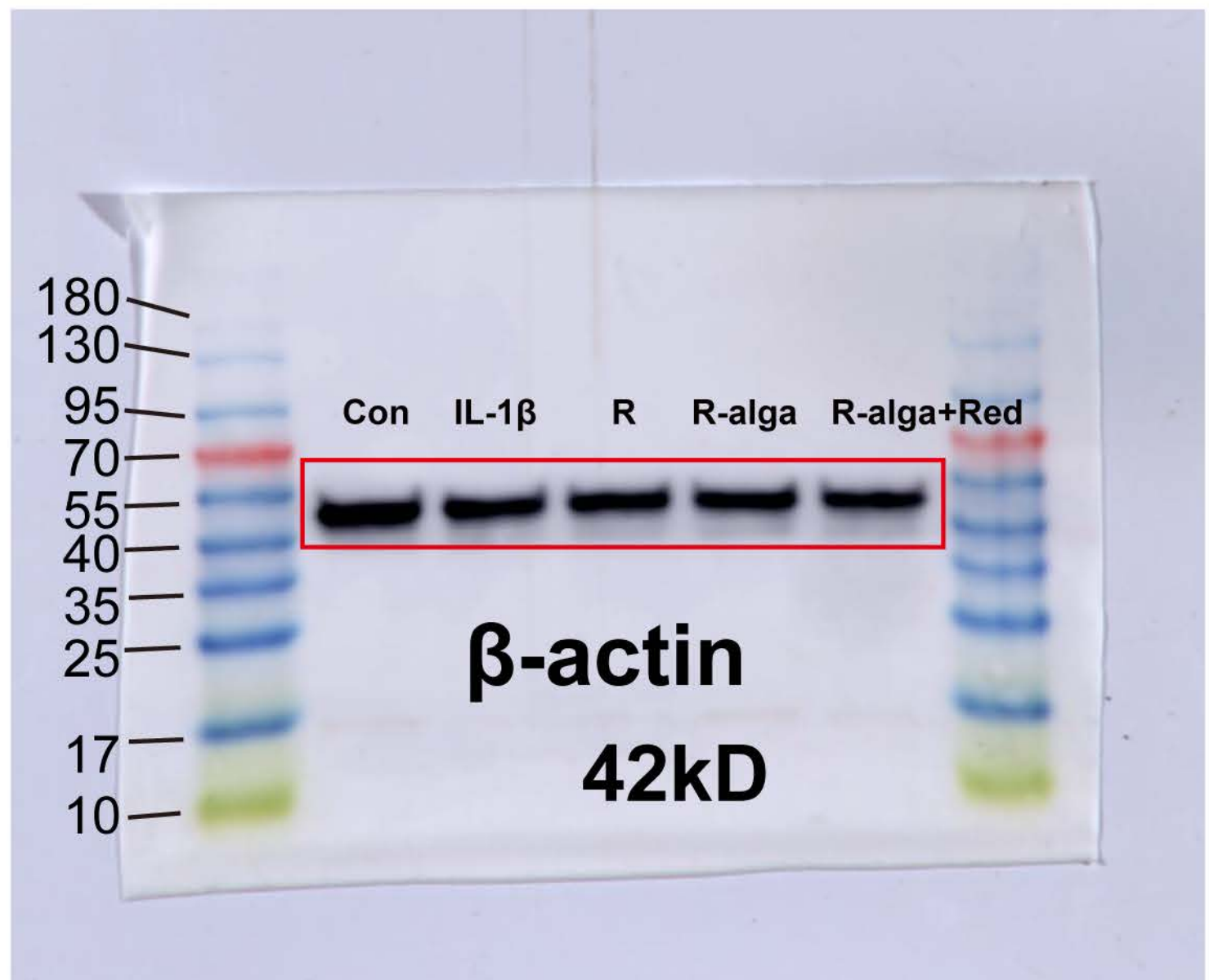


# Repeat 3

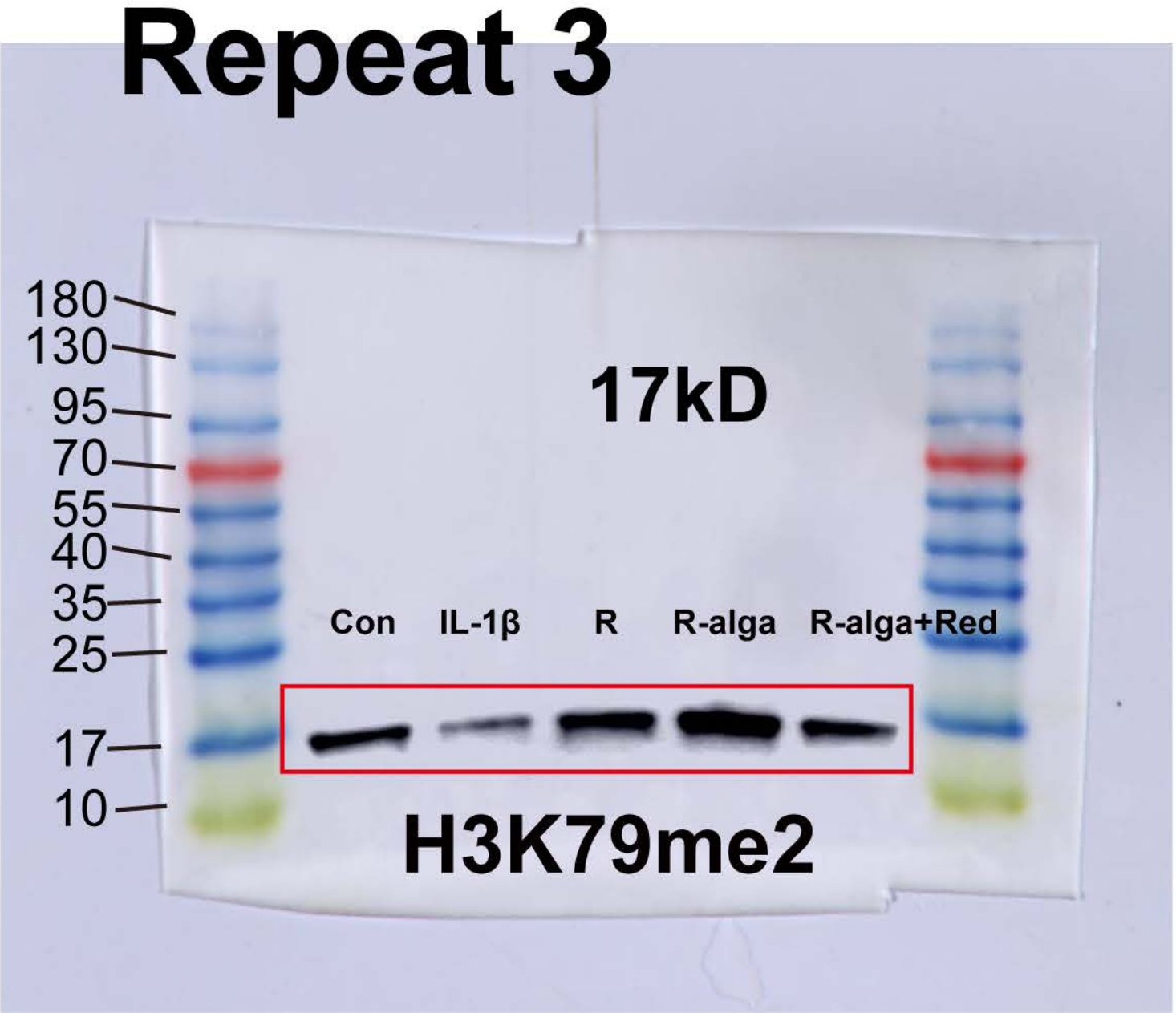


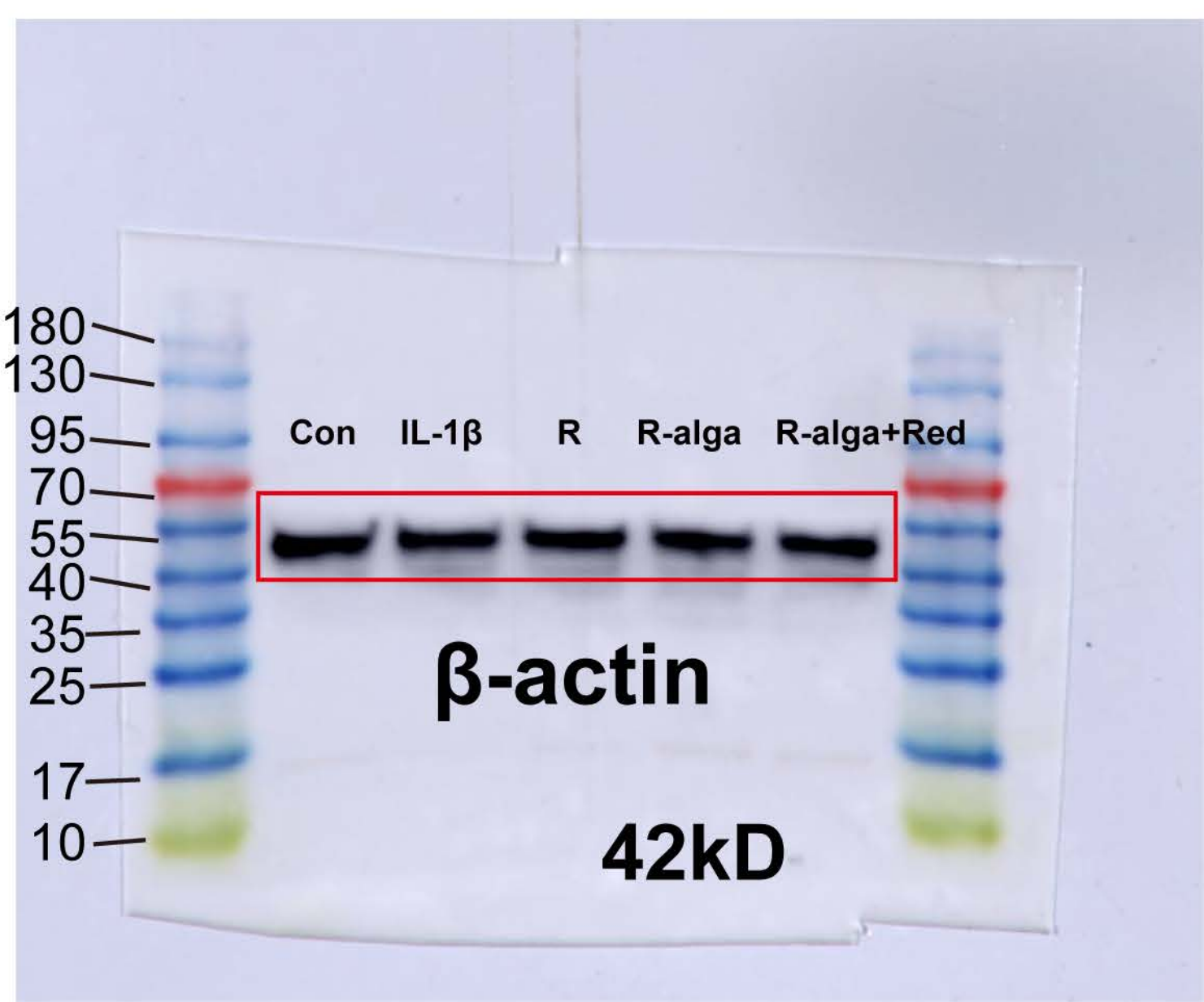

# Repeat 1

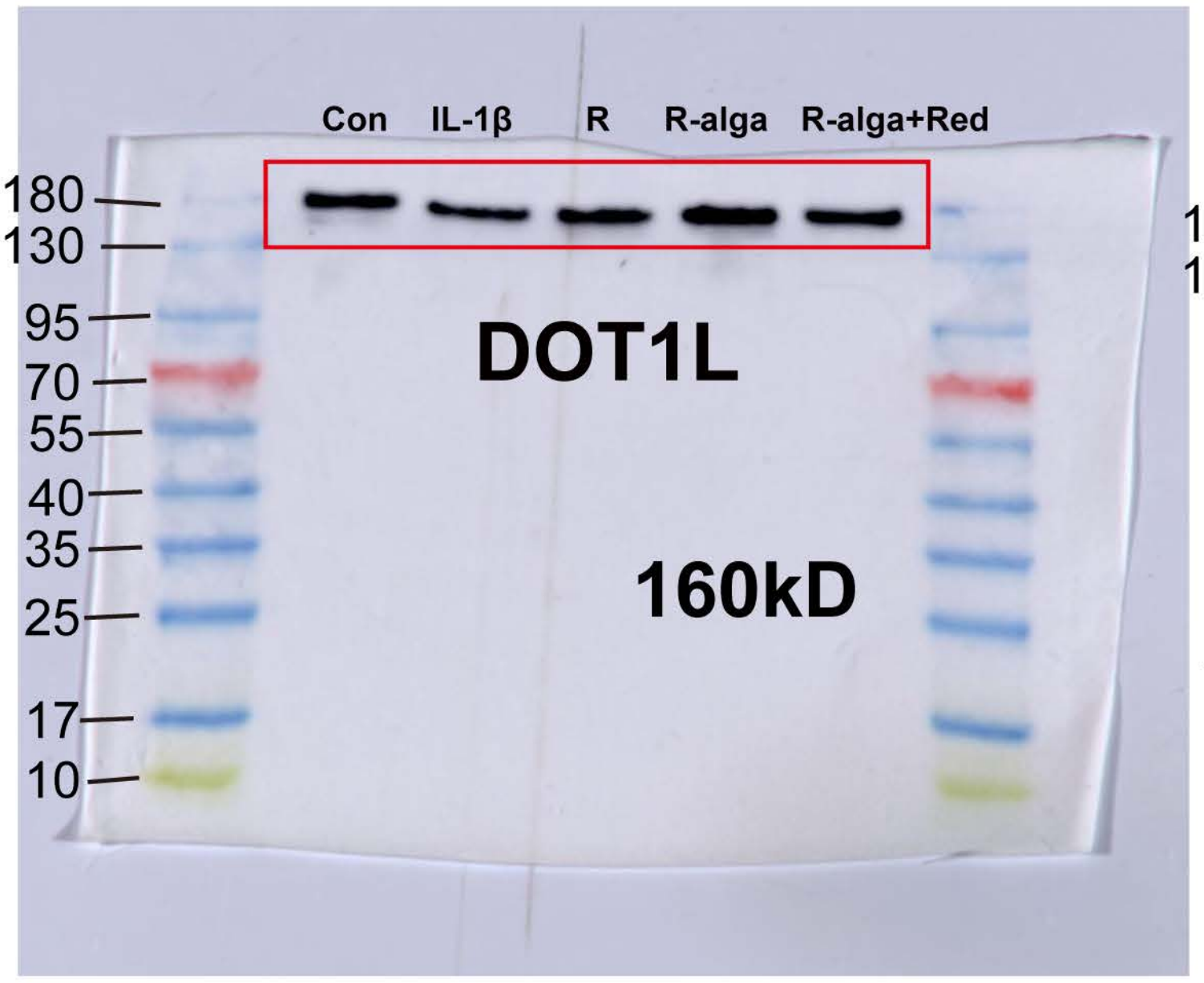


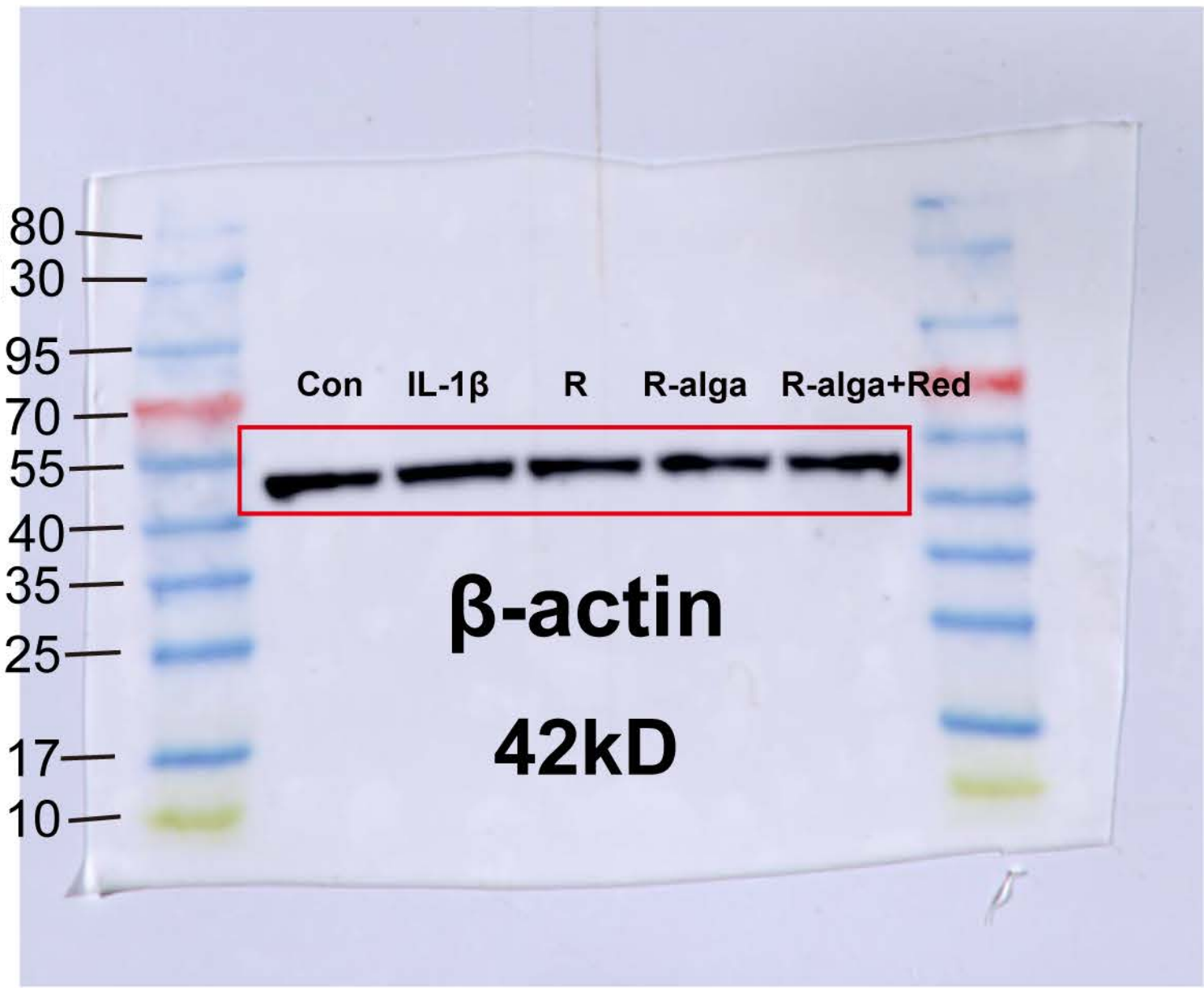


# Repeat 2

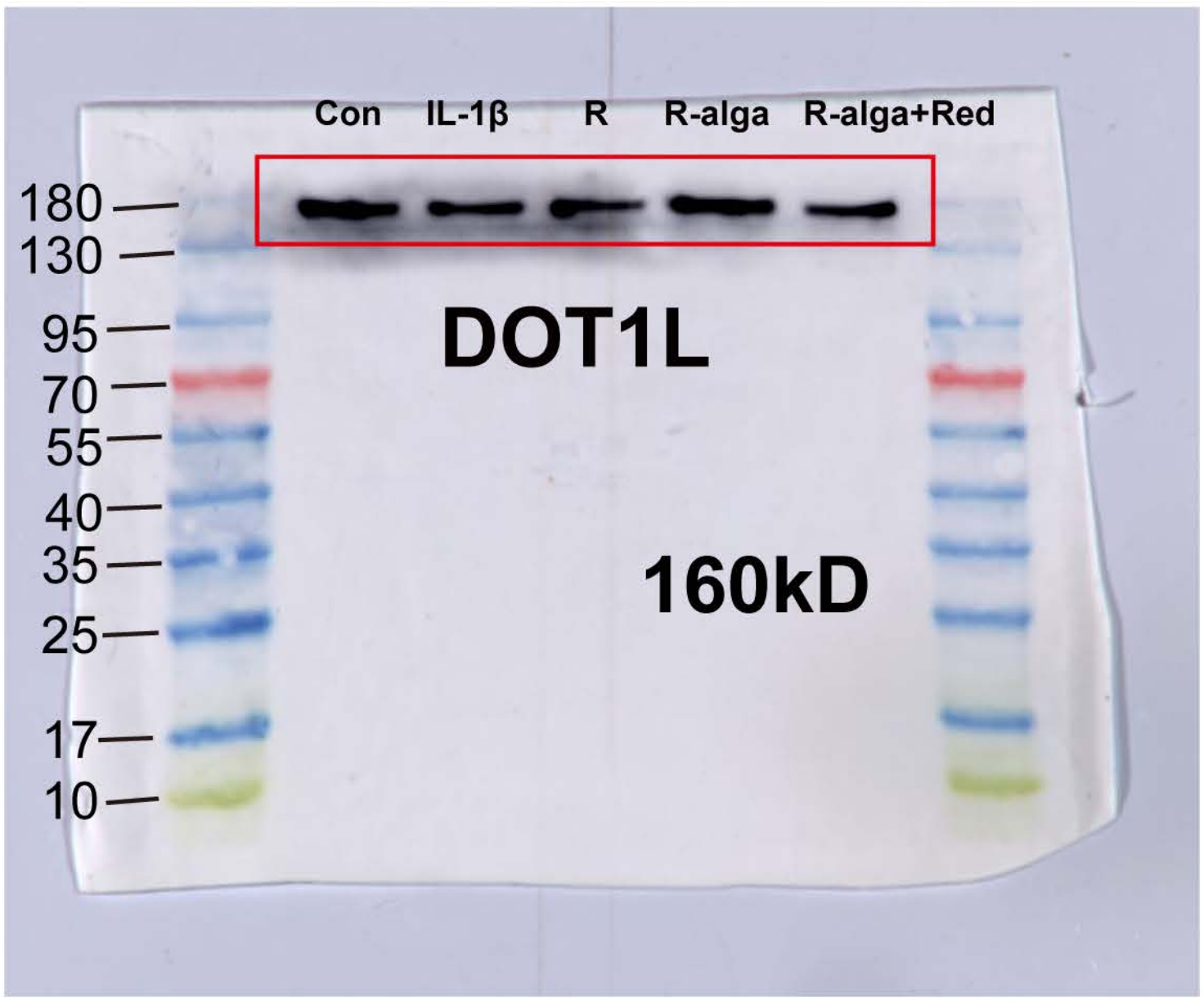


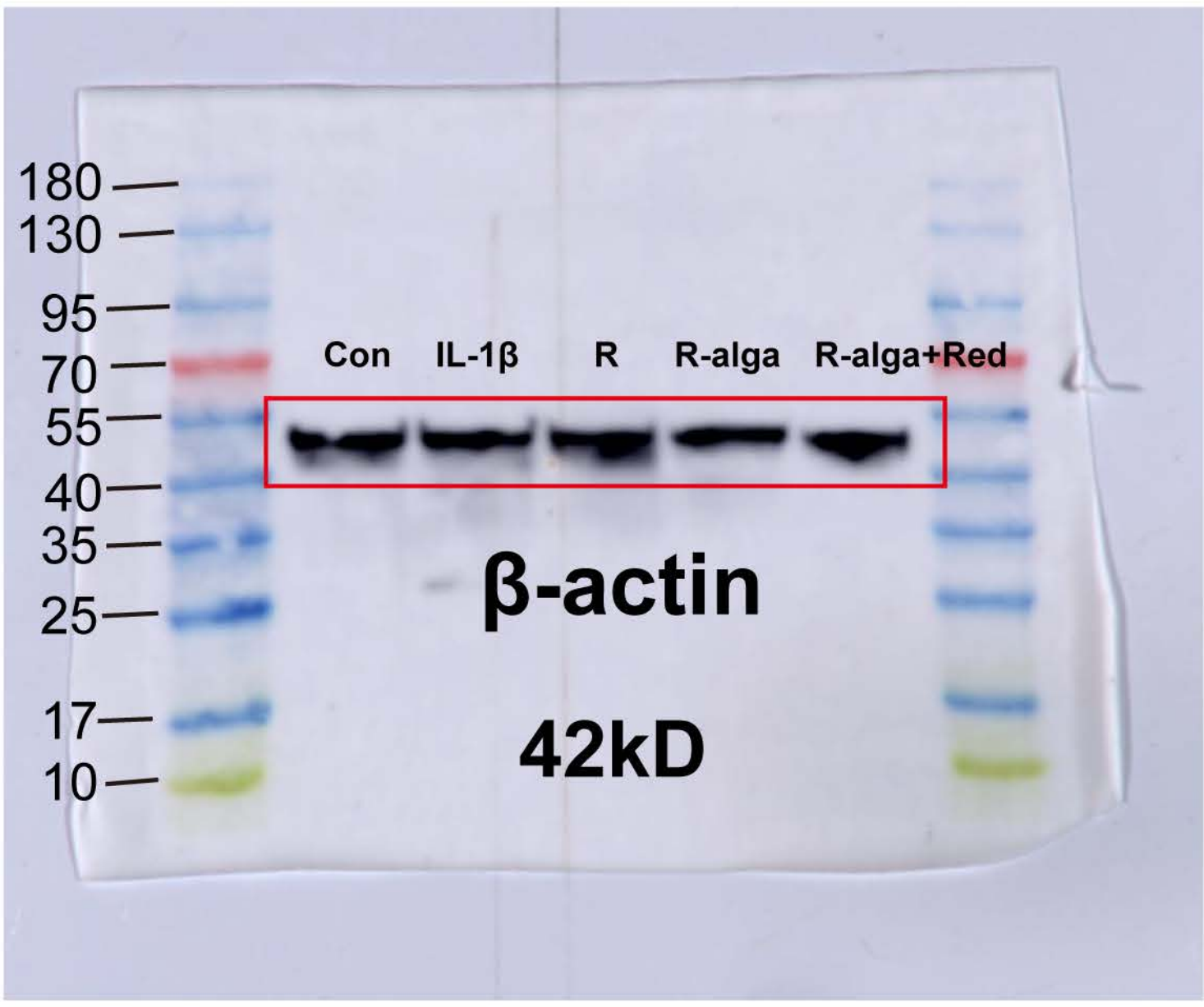


# Repeat 3

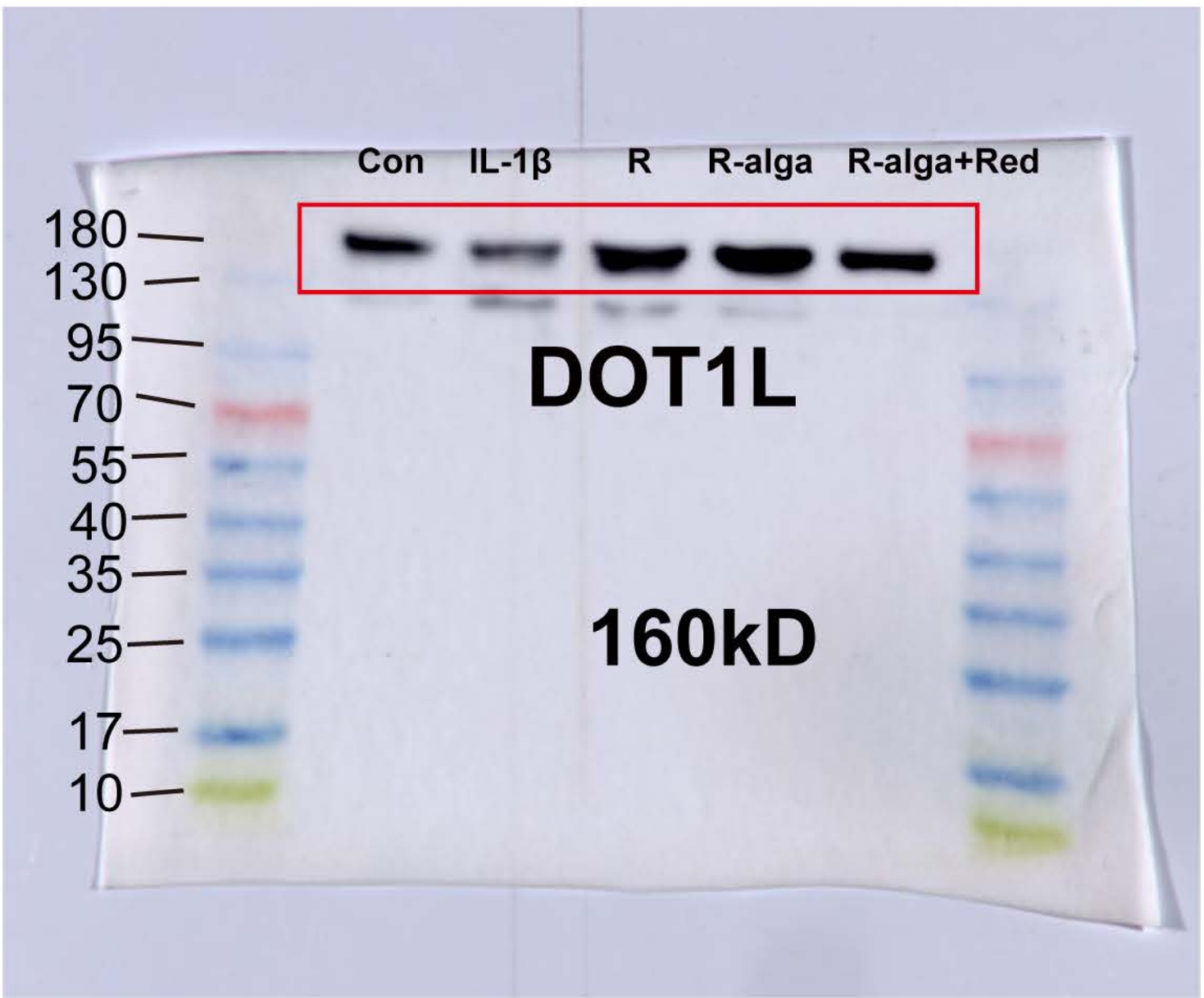


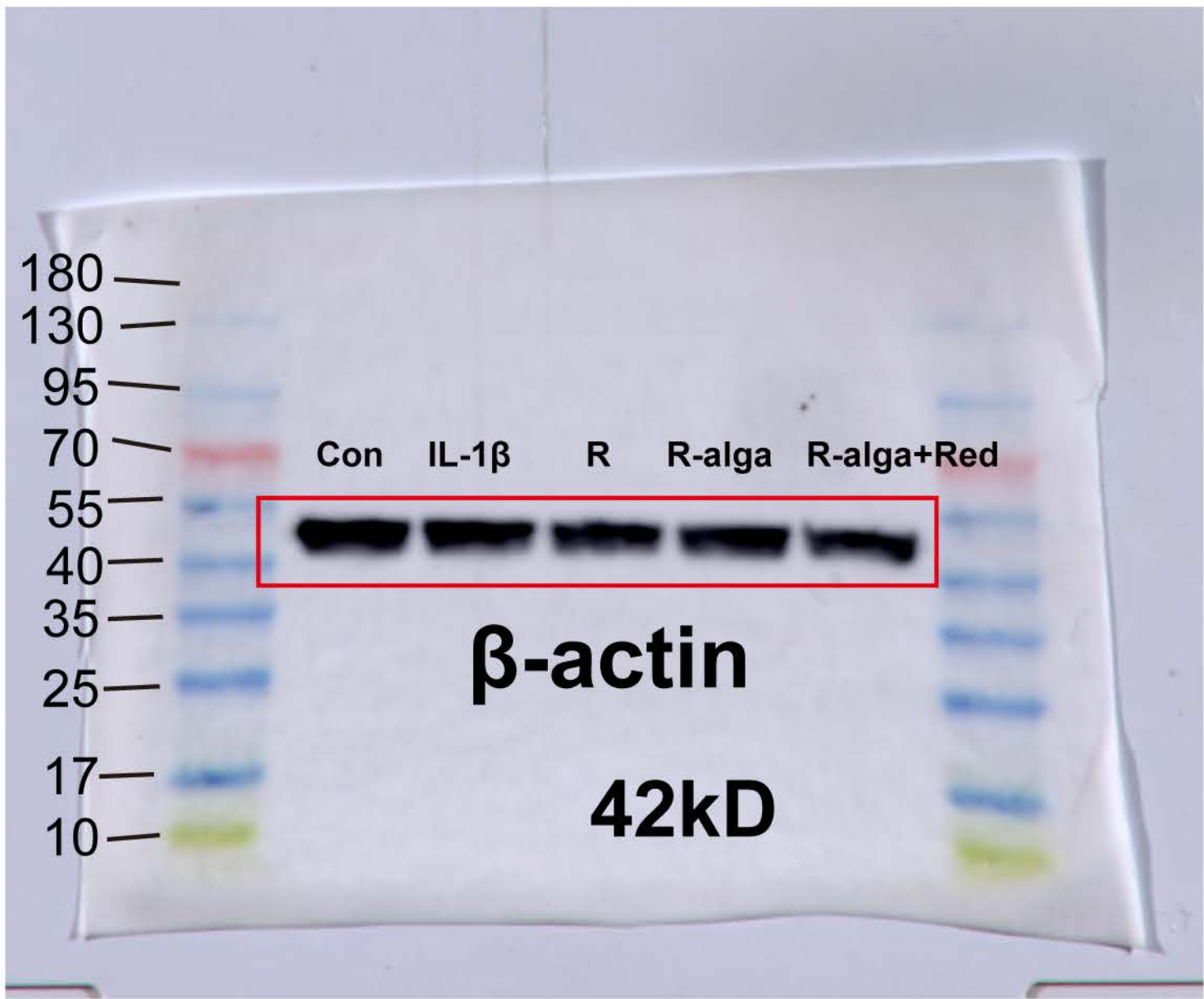

Supplement: Supplementary file 1 [file ADVS-9999-e77248-s001.pdf]
