# Supplementary material for: Engineered Cyanobacteria‐Hydrogel Microecological System for Oxygen Reprogramming Synergizes Immunomodulation With Anabolism in Osteoarthritis Therapy
Source: Adv Sci (Weinh). 2026 Aug 31:e77248. Online ahead of print. doi: 10.1002/advs.77248 (PMC13528180; doi:10.1002/advs.77248)
Supplement: Supplementary file 2 [file ADVS-9999-e77248-s002.docx]

1. Cell source and procurement information

Primary rat articular chondrocytes were isolated from the articular cartilage of 1-week-old male Sprague-Dawley (SD) rats. Cells were cultured in DMEM medium (Gibco, USA) supplemented with 10% fetal bovine serum (FBS, Gibco, USA) and 1% penicillin-streptomycin (Gibco, USA), and maintained at 37 °C in a humidified incubator with 5% CO₂. Cells at passage 2 (P2) were used for all *in vitro* experiments to ensure cell viability and phenotypic stability.

RAW 264.7 murine macrophages were purchased from servicebio (Cat. No. STCC20020，RRID:CVCL_0493). The cell line was cultured in high-glucose DMEM medium (Gibco, USA) supplemented with 10% fetal bovine serum (FBS, Gibco, USA) and 1% penicillin-streptomycin (Gibco, USA), and maintained at 37 °C in a humidified incubator with 5% CO₂. Cells were passaged every 2-3 days when reaching 80-90% confluency, and cells at passages 3-5 (P3-P5) were used for *in vitro* experiments to ensure cell activity and stable phenotypic

2. Antibody RRID information

IL-6 antibody (Cat. No. 21865-1-AP, Proteintech, Wuhan, CN; RRID:AB_11142677)

COL2A1 antibody (Cat. No. 28459-1-AP, Proteintech, Wuhan, CN; RRID: AB_2881147)

MMP13 antibody (Cat. No. 18165-1-AP, Proteintech, Wuhan, CN; RRID: AB_2144858)

HIF-1α antibody (Cat. No. 20960-1-AP, Proteintech, Wuhan, CN; RRID: AB_10732601)

Lubricin (PRG4) antibody (Cat. No. 25732-1-AP, Proteintech, Wuhan, CN; RRID: AB_2880215)

CD86 antibody (Cat. No. 83213-1-RR, Proteintech, Wuhan, CN; RRID:AB_3670897)

CD206 antibody (Cat. No. 83485-1-RR, Proteintech, Wuhan, CN; RRID: AB_3671113)

H3K79me2 antibody (Cat. No. DF6946, Affinity, Cincinnati, USA; RRID: AB_[2838905](https://antibodyregistry.org/search.php?q=AB_2838905))

DOT1L antibody (Cat. No.	DF2490, Affinity, Cincinnati, USA; RRID: AB_[2839696](https://antibodyregistry.org/search.php?q=AB_2839696))

F4/80 antibody (Cat. No. 28463-1-AP, Proteintech, Wuhan, CN; RRID: AB_2881149)

PHGDH antibody (Cat. No. DF12220, Affinity, Cincinnati, USA; RRID: AB_[2845025](https://antibodyregistry.org/search.php?q=AB_2845025))

PSAT1 antibody (Cat. No.	DF12132, Affinity, Cincinnati, USA; RRID: AB_[2844937](https://antibodyregistry.org/search.php?q=AB_2844937))

HIF-3α antibody (Cat. No. DF8557, Affinity, Cincinnati, USA; RRID: AB_[2841761](https://antibodyregistry.org/search.php?q=AB_2841761))

PE anti-mouse CD86 antibody (Cat. No. 561963 BD Biosciences, USA; RRID:AB_10896971);

Fitc Rat Anti CD11b antibody (Cat. No. 561688 BD Biosciences, USA; RRID:AB_10898180)

FITC-conjugated goat anti-rabbit IgG secondary antibody (Cat. No. SA00003-2, Proteintech, Wuhan, CN; RRID:AB_2890897)

HRP-conjugated goat anti-rabbit IgG secondary antibody (Cat. No. RGAR301, Proteintech, Wuhan, CN; RRID:AB_3712952)

3. Software used in the experiment

ImageJ software (National Institutes of Health, Bethesda, MD, USA; RRID:SCR_003070) was used for quantitative analysis of immunofluorescence intensity, live/dead staining cell counting, and Western blot band gray value quantification.

GraphPad Prism 9 (GraphPad Software, San Diego, CA, USA; RRID:SCR_002798) was used for statistical analysis and graph generation.

SPSS 26.0 (IBM Corp., Armonk, NY, USA; RRID:SCR_002865) was used for statistical tests to determine significant differences between groups.
