# Supplementary material for: Engineered Cyanobacteria‐Hydrogel Microecological System for Oxygen Reprogramming Synergizes Immunomodulation With Anabolism in Osteoarthritis Therapy
Source: Adv Sci (Weinh). 2026 Aug 31:e77248. Online ahead of print. doi: 10.1002/advs.77248 (PMC13528180; doi:10.1002/advs.77248)
Supplement: Supplementary file 3 [file ADVS-9999-e77248-s003.doc]

**Engineered Cyanobacteria-Hydrogel Microecological System for Oxygen Reprogramming Synergizes Immunomodulation with Anabolism in Osteoarthritis Therapy**

Lerong Yang^1,2#^, Chong Shen^2#^, Hongjun Huang^1#^, Siyi Liu^1,3^, Zeyu Huang^1^, Licheng Xi^2^, Huan Cao^4*^, Li Zheng^1*^, Zainen Qin^1*^

^1^Guangxi Key Laboratory of Regenerative Medicine, Guangxi Engineering Center in Biomedical Materials for Tissue and Organ Regeneration, International Joint Laboratory on Regeneration of Bone and Soft Tissues, Collaborative Innovation Center of Regenerative Medicine and Medical Bioresource Development and Application Co-constructed by the Province and Ministry, The First Affiliated Hospital of Guangxi Medical University, Guangxi Medical University, Nanning 530021, China

^2^Department of Orthopaedics, Affiliated Hospital of Guilin Medical University, Guilin, Guangxi 541000, China

^3^Life Sciences Institute, Guangxi Medical University, Nanning, 530021, China

^4^National Engineering Research Center for Biomaterials, College of Biomedical Materials, Sichuan University, Chengdu 610064, China

^#^These authors contributed as the first authors.

Corresponding authors.

E-mail addresses: [zhengli224@163.com](mailto:zhengli224@163.com); [caoh0005@scu.edu.cn](mailto:caoh0005@scu.edu.cn;); qn78495036@126.com

**Figure S1**. Fourier infrared spectra of different materials.

**Figure S2**. Storage modulus and Loss modulus of R-alga hydrogel with strain variation.

**Figure S3**. Variation of complex viscosity of R-alga over time.

**Figure S4**. Typical compression strain-stress curve of R-alga hydrogel.

**Figure S5**. Quantification of oxygen levels under red light irradiation and darkness conditions, respectively.

**Figure S6**. The change of absorbance intensities at 680 nm of cyanobacteria within R-alga hydrogels at different days. Data were presented as mean ± SD, n = 3 independent samples.

**Figure S7**. Bright field, fluorescence and merged images of cyanobacteria within R-alga hydrogels after different days under physiological conditions. The results were representative of three independent experiments. Scale bars represent 200 μm.

**Figure S8**. Chondrocytes and RAW264.7 macrophage metabolic activity measured by CCK-8 assay after being cocultured with hydrogels. Data were presented as mean ± SD, n = 3 independent samples.

**Figure S9**. Quantification of Chondrocyte and RAW264.7 cell number after being cocultured with hydrogels. Data were presented as mean ± SD, n = 3 independent samples.

**Figure S10**. Live/dead staining of chondrocytes (a) and macrophages (b) after being co-cultured with hydrogel. Scale bars represent 400 μm.

**Figure S11**. Fluorescence images of ROS staining (a) and corresponding flow cytometry analyses (b) in cartilage cells after different treatments. Scale bars represent 200 μm.

**Figure S12.** Representative images of [Ru(dpp)_3_]Cl₂ in macrophages subjected to various treatments, all under hypoxic conditions.

**Figure S13**. Quantification of CD86+ cells number in the population after different treatment. ****p* <0.001.

**Figure S14.** Representative images of oxygen indicator [RU (DPP)_3_] Cl_2_ in cartilage cells after different treatments. Scale bars represent 200 μm.

**Figure S15.** Immunofluorescence staining and corresponding statistical results of PRG4 expression in chondrocytes after different treatments. Scale bars represent 200 μm. Data are expressed as mean ± SD (n = 3). *p <0.05, **p <0.01, ***p <0.001.

**Figure S16**. Frozen sections of rat joints at 12 hours (a) and 3 days (b) after intra-articular injection of hydrogel. Red represents cyanobacteria and green represents hydrogel. Scale bars represent 100 μm.

**Figure S17**. The expression levels of IL-1β (a), TNF-α (b), and IL-6 (c) in joint fluid; (d) Histological evaluation of synovial membrane morphology by H&E staining in rats subjected to different treatments (Ctrl vs. R-alga + Red). (e-i) Changes in complete blood cell count and blood biochemical parameters of rats after different treatments. Data are expressed as mean ± SD (n = 3). ns indicates no significant difference (p > 0.05).

**Figure S18**. (a) Immunohistochemical quantitative analysis of synovial IL-6 expression; (b) fluorescence quantitative analysis of M1 macrophage proportion, and (c) fluorescence quantitative analysis of synovial HIF-1α expression. Data are expressed as mean ± SD (n = 5). **p* <0.05, ***p* <0.01, ****p* <0.001.

**Figure S19**. Immunohistochemical quantitative analysis of cartilage IL-6 (a) and MMP13 (b) expression, fluorescence quantitative analysis of cartilage COL2(c) and HIF-1α(d) expression. Data are expressed as mean ± SD (n = 5). **p* <0.05, ***p* <0.01, ****p* <0.001.

**Figure S20**. KEGG enrichment analysis of cartilage transcriptome.

**Figure S21**. Immunohistochemistry analyses of phgdh, psat1, and h3k79me2 after different treatments.

**Table 1** Primer sequences for qRT-PCR

| Gene name | Forward primers | Reverse primers |
| --- | --- | --- |
| CD86-M | 5’-CTCAGATGCTGTTTCCGTGG-3’ | 5’-CTGTGCÇCAAATAGTGCTCG-3’ |
| CD206-M | 5’-AGGGTGCGGTACACTAACTG-3’ | 5’-TCTGACTCTGGACACTTGCC-3’ |
| TNF-α-M | 5’-CAGAAAGCATGATCCGCGAC-3’ | 5’-ATCCCTTTGGGGACCGATCA-3’ |
| IL-6-M | 5’-GGAGCCCACCAAGAACGATAG-3’ | 5’-GTGAAGTAGGGAAGGCCGTG-3’ |
| TGF-β-M | 5’-GTCAGACATTCGGGAAGCAG-3’ | 5’-AGCCACTCAGGCGTATCAGT-3’ |
| GADPH-M | 5’-ACTTGAAGGGTGGAGCCAAA-3’ | 5’-GCCCTTCCACAATGCCAAAG-3’ |
| DOT1L-R | 5’-TGGAAAATGGTGGTGGCTTAG-3’ | 5’-CGAGGTCGGAGATGGGTGA-3’ |
| HIF-1α-R | 5’-TTGCATCTCCACCTTCTACCC-3’ | 5’-TGTCGGTGAGGTTGTCCGA-3’ |
| COL2A1-R | 5’-ATTGCCTACCTGGACGAAGC-3’ | 5’-GACAGGCCCTATGTCCACAC-3’ |
| ANCA-R | 5’-GACAAGGACGAGTTCCCTG-3’ | 5’-CTCCGGGGATGTGGCATAAA-3’ |
| NFKB-R | 5’-TGTGGGGTTTCAGGATAA-3’ | 5’-TGGATGATGGCTAAGTGT-3’ |
| MMP-13-R | 5’-GCCATTACCAGTCTCCGAGG-3’ | 5’-TACGGTTGGGAAGTTCTGGC-3’ |
| IL-6-R | 5’-GAGCCCACCAAGAACGATAG-3’ | 5’-ACAGTGCATCATCGCTGTTC-3’ |
| Sox9-R | 5’-AGTACCCGCATCTGCACAAC-3’ | 5’-ACGAAGGGTCTCTTCTCGCT-3’ |
| GADPH-R | 5’-AGGGCCCTGACAACTCTTTT-3’ | 5’-AGGGGTCTACATGGCAACTG-3’ |
